# Systematic investigation on transverse thermoelectric conversion of RE_2_(Fe,Co)_14_B (RE = rare-earth) compounds

**DOI:** 10.1080/14686996.2025.2520162

**Published:** 2025-06-18

**Authors:** Babu Madavali, Fuyuki Ando, Takamasa Hirai, Andres Martin-Cid, Ken-ichi Uchida, Hossein Sepehri-Amin

**Affiliations:** a Research Center for Magnetic and Spintronic Materials, National Institute for Materials Science, Tsukuba, Japan; b Department of Advanced Materials Science, Graduate School of Frontier Sciences, The University of Tokyo, Kashiwa, Japan

**Keywords:** Anomalous Nernst effect, permanent magnets, transverse thermoelectric conversion

## Abstract

Transverse thermoelectric generation (TEG) based on the anomalous Nernst effect (ANE) is a promising technology capable of converting waste heat into electricity. The transverse TEG device can operate without applying an external magnetic field by implementing permanent magnets, such as Nd_2_Fe_14_B-type magnets. However, the transverse thermoelectric properties of the rare-earth (RE) based compounds have not been systematically studied and understood so far. In this work, we have explored the potential of RE_2_Fe_14_B and Nd_2_(Fe, Co)_14_B compounds and systematically investigated the transverse thermoelectric properties at room temperature. We obtained the negative anomalous Nernst coefficient (*S*_ANE_) for all the RE_2_Fe_14_B (RE = Tb, Dy, Ho, and Nd) alloys regardless of the RE element and the highest negative *S*_ANE_ of −0.67× 10^−6^ VK^−1^ for Tb_2_Fe_14_B among them, which is comparable to that of the commercial Nd_2_Fe_14_B permanent magnets with an optimized microstructure. The substitution of Co for Fe site in Nd_2_(Fe_1-*p*_Co_*p*_)_14_B alloys causes sign reversal (from negative to positive) of *S*_ANE_ values. The transverse thermoelectric conductivity is responsible for the sign change in *S*_ANE_ values. As a result, the Nd_2_(Fe_0.4_Co_0.6_)_14_B alloy shows the highest positive *S*_ANE_ of 1.87 × 10^−6^ VK^−1^, revealing that the RE and 3*d* transition metal elements play distinct roles on the transverse thermoelectric performance in RE_2_(Fe,Co)_14_B compounds.

## IMPACT STATEMENT

This work systematically investigates rare-earth-based RE₂Fe₁₄B compounds, uncovering tunable transverse thermoelectric properties, including anomalous Nernst coefficient sign reversal and a twofold enhancement via Co substitution, advancing efficient transverse thermoelectric generation technologies.

## GRAPHICAL ABSTRACT

**Figure:**
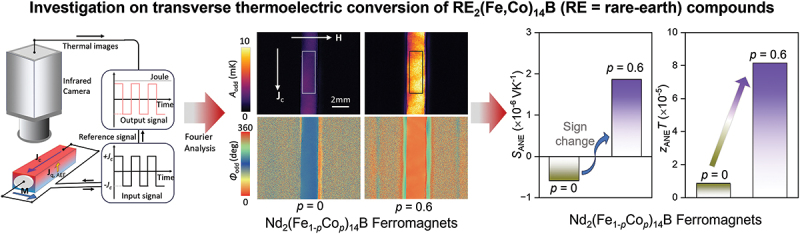

## 1. Introduction

Transverse thermoelectric generation (TEG) has garnered significant attention due to its ability to directly convert heat into electricity [1,2]. A typical TEG device based on the Seebeck effect consists of multiple *p*-type and *n*-type semiconductor legs, connected electrically in series and thermally in parallel using a large number of electrodes. However, such longitudinal TEG devices face unique challenges: thermal degradation at the hot side of the contacts, which significantly deteriorates the devices’ output power, and performance degradation due to interfacial thermal and electrical resistances at many junctions [1,3,4]. In this context, transverse TEG devices offer key advantages over longitudinal TEG devices due to their simpler structures, which enables less junctions and electrodes in modules. Consequently, these devices avoid the above degradations, significantly increasing efficiency closer to the theoretical limit than the conventional TEG devices [3–5].

**Figure 1.**
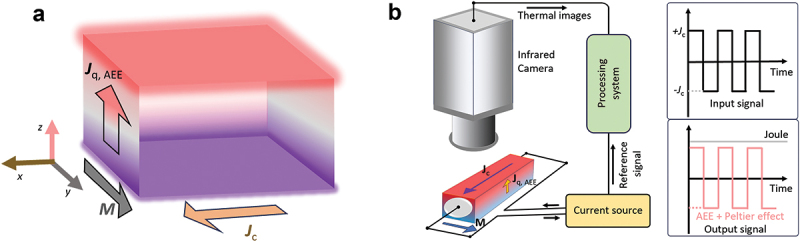
(a) Schematic of AEE in a ferromagnet in the in-plane magnetization configuration. $j_{q,\mathrm{AEE}}$, $j_{c}$, and **M** denote the AEE-induced heat current, charge current applied to the ferromagnet, and magnetization vector. (b) Schematic of the LIT measurement set-up configurations. *J*_c_ denotes the square-wave amplitude of the charge current applied to the sample.

In various transverse thermoelectric phenomena, the anomalous Nernst effect (ANE) has received much interest because of its simple lateral device structures and its intriguing physical mechanisms [6–18]. ANE refers to the generation of an electric field (**E**_ANE_) orthogonal to the magnetization direction and applied temperature gradient (∇*T*) in magnetic materials:

(1) $$E_{\mathrm{ANE}}=S_{\mathrm{ANE}}\left(m\times \nabla T\right)$$

where **m** is a unit vector of the magnetization. The anomalous Ettingshausen effect (AEE) is the reciprocal to ANE in which the transverse heat current is generated in the direction orthogonal to the magnetization direction and applied charge current in magnetic materials, shown in Figure 1(a) [19–26]. The heat current density driven by AEE ($j_{q,\mathrm{AEE}}$) is defined as

(2) $$j_{q,\mathrm{AEE}}=\Pi_{\mathrm{AEE}}\left(j_{c}\times m\right)$$

where **j**_c_ and $\Pi_{\mathrm{AEE}}$ respectively denote the charge current density and anomalous Ettingshausen coefficient ($\Pi_{\mathrm{AEE}}=S_{\mathrm{ANE}}T$) [23,25–27]. Based on Equations 1 and 2, a finite spontaneous magnetization is required in materials for the manifestation of the ANE/AEE in transverse thermoelectric applications. Although elemental transition metals (e.g. Fe, Co) exhibit trivial ANE/AEE, recent development of spin caloritronics and topological materials science has revealed several magnetic alloys with large $S_{\mathrm{ANE}}$ and $\Pi_{\mathrm{AEE}}$ [6,7,11,15,16,27–30]. For example, Sakai et al. observed giant ANE in Co_2_MnGa alloys with *S*_ANE_ ~6 × 10^−6^ VK^−1^ at room temperature by focusing on the Berry curvature of conduction electrons around Fermi energy [28]. Gautam et al. reported another strategy of enhancing ANE/AEE from the viewpoint of nanostructure engineering using iron-based amorphous alloys [30]. However, most of the ANE-based TEG devices using these materials require an external magnetic field (*H*) to align the magnetization direction.

To solve this problem, the use of permanent magnets with large remanent magnetization in ANE-based TEG devices is useful, enabling their thermoelectric operation without applying an external magnetic field [4,25,26]. Miura et al. investigated the transverse thermoelectric properties of different commercial sintered magnets and reported SmCo_5_-type (Nd_2_Fe_14_B-type) permanent magnets under zero magnetic fields give the maximum *S*_ANE_ of 3.10 × 10^−6^ VK^−1^ (−0.76 × 10^−6^ VK^−1^) at room temperature [25]. Recently, Ando et al. constructed a bulk TEG device consisting of these SmCo_5_- and Nd_2_Fe_14_B-type permanent magnets and demonstrated the largest anomalous Nernst power density of 65 μWcm^−2^ under zero magnetic field [4]. However, the research so far on transverse thermoelectric properties of bulk permanent magnets has been focused on the commercially available magnets designed for the maximum energy product. Development of permanent magnets with excellent transverse thermoelectric properties requires fundamental research on their alloy and microstructure design.

In this work, we have systematically studied the transverse thermoelectric properties of RE_2_Fe_14_B-type compounds by changing the RE (RE = Tb, Dy, Ho, and Nd) elements. The *f*-electron system was chosen to make the compounds that exhibit strong magnetocrystalline anisotropy, particularly due to the RE – Fe exchange interactions and crystal electric field effects on the RE site [31]. This magnetic order affects charge carrier dynamics and thermal transport, which may favor the improvement in transverse thermoelectric properties. We found that all these compounds show negative *S*_ANE_ regardless of RE elements, and Tb_2_Fe_14_B exhibits the largest *S*_ANE_ among them. In addition, we found that substitution of Co for the Fe site in the Nd_2_Fe_14_B system can change the sign of *S*_ANE_; from negative in Nd_2_Fe_14_B to positive in Nd_2_Co_14_B. At the intermediate composition for Nd_2_(Fe_0.4_Co_0.6_)_14_B, the largest *S*_ANE_ of 1.87 × 10^−6^ VK^−1^ was observed. This study provides guidelines for the alloy design of permanent magnets for the development of transverse TEG devices.

## 2. Materials and methods

A series of RE_2_Fe_14_B (RE = Tb, Dy, Ho, and Nd) and Nd_2_(Fe_1-*p*_Co_*p*_)_14_B (*p* = 0, 0.2, 0.4, 0.6, 0.8, and 1.0) alloys were prepared by an induction melting process under an Ar atmosphere. The as-cast alloys were homogenized at 1150ºC for 24 h in high vacuum conditions and quenched in water. The phase analysis of the ingots was evaluated by X-ray diffraction (XRD) (MiniFlex600, Rigaku) with Cr-Kα radiation. The XRD patterns were analyzed by the Rietveld method using the FullProf Suite [32]. Microstructural and chemical analyses were performed using a scanning electron microscope (SEM) (CrossBeam 1540 EsB, Carl Zeiss) equipped with an energy-dispersive X-ray spectroscopy (EDS) detector.

To investigate the transverse thermoelectric properties of RE_2_(Fe,Co)_14_B alloys, we performed AEE using the lock-in thermography (LIT) technique using Enhanced Lock-In Thermal Emission (ELITE, DCG Systems G.K.) (Figure 1(b)) [23,25–27]. AEE measurements were conducted on rectangular-shaped samples (width and thickness of ~2–3 mm and a length of ~15 mm). The samples were placed on a plastic slab, which has a low thermal conductivity to reduce the heat loss due to thermal conduction. To enhance the infrared emissivity and ensure the homogenous emission properties, the top surface of the samples was coated with a black ink, the emissivity of which is >0.94 (JSC-3, JAPANSENSOR Corporation). The AEE measurements were conducted under the application of magnetic field, *µ*_0_*H* = 1 T, with an atmospheric temperature and pressure. The thermal images were attained while applying a square-wave-modulated charge current with an amplitude *J*_c_, frequency *f*, and zero offset to the sample along the *x*-direction and *H* along the *y*-direction. By extracting the first-harmonic component of the temperature modulation signal, the contributions of thermoelectric response, i.e. AEE and the Peltier effect, free from Joule heating, can be detected because Joule heating generated by such a charge current is constant over time [19,20,22,25]. The detected thermal images are transformed into the lock-in amplitude *A* and phase ϕ via Fourier analysis, where the *A* image gives the distribution of the magnitude of temperature modulation signals and the ϕ image shows the sign of the temperature modulation as well as the time delay due to thermal diffusion. By performing the LIT measurements under positive and negative *H* and evaluating the *H*-odd component of *A* and ϕ (*A*_odd_ and ϕ_odd_, respectively), we can visualize the distribution of AEE-induced temperature modulation signals without the parasitic contribution by the Peltier effect [4,27,30]. In the present study, LIT measurements were performed at *µ*_0_*H* = ± 1 T. Since the present compounds are isotropic ferromagnetic ingots, it is necessary to accurately evaluate the magnetization at *µ*_0_*H* = 1 T, *M*_1T_, by taking into account the shape magnetic anisotropy. Therefore, we carefully determine *M*_1T_ using the same size Ni slab as a reference sample by vibrating sample magnetometry (VSM) and the same slabs used for the AEE measurement. The measurements of the anomalous Hall effect (AHE) were performed to estimate the transverse electrical resistivity *ρ*_*xy*_ using a physical property measurement system (PPMS, Quantum Design, Inc.). For this measurement, we applied *H* perpendicular to a DC charge current of 100 mA. The Seebeck coefficient *S*_*xx*_ and electrical conductivity *σ* of the samples were measured by Seebeck Coefficient/Electric Resistance Measurement System (ZEM-3, ADVANCE RIKO, Inc.). The thermal diffusivity measurements using the laser flash method and the specific heat measurements using the differential scanning calorimetry were performed to evaluate the thermal conductivity *κ*.

## 3. Results and discussion

### 3.1. Structural analysis of RE_2_Fe_14_B alloys

**Figure 2.**
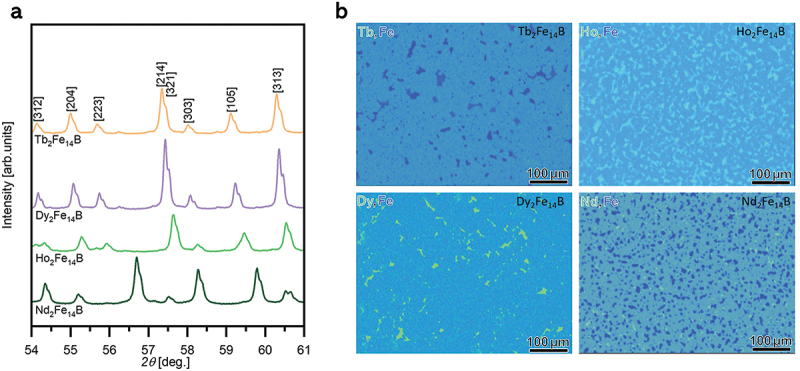
(a) Crystal structure analysis using XRD and (b) chemical composition analysis using SEM-EDS for the RE_2_Fe_14_B alloys.

The crystal structure and chemical composition analyses of RE_2_Fe_14_B alloys are depicted in Figure 2. The XRD patterns were analyzed through Rietveld refinement, identifying the polycrystalline 2:14:1 phase as the main phase and indexed with (*hkl*) planes accordingly. The detailed Rietveld refinement analysis of all RE_2_Fe_14_B alloys is shown in supplementary Figures S1–S4 and where several secondary phases were identified in addition to the main phase. The identified secondary phases include bcc-Fe, NdFe_4_B_4_, TbFe_2_, HoFe_3_, Ho_6_Fe_23_, and DyFe_2_ and represent <10% in weight of the alloy phases [33,34]. The detailed microstructure characterization using SEM-EDS gives evidence that the samples are mainly made of 2:14:1 phase polycrystals as the matrix phase with homogeneously dispersed secondary phases (Figure 2(b)). The secondary phases identified by XRD can be correlated to the different composition regions found in the SEM-EDS images, as examples are shown in supplementary Figures S1–S4. However, the identification of all secondary phases in the Ho_2_Fe_14_B system remains challenging, and some of the secondary reflections found in XRD would not be identified with the existing phases.

### 3.2. Transverse thermoelectric properties of RE_2_Fe_14_B alloys

**Figure 3.**
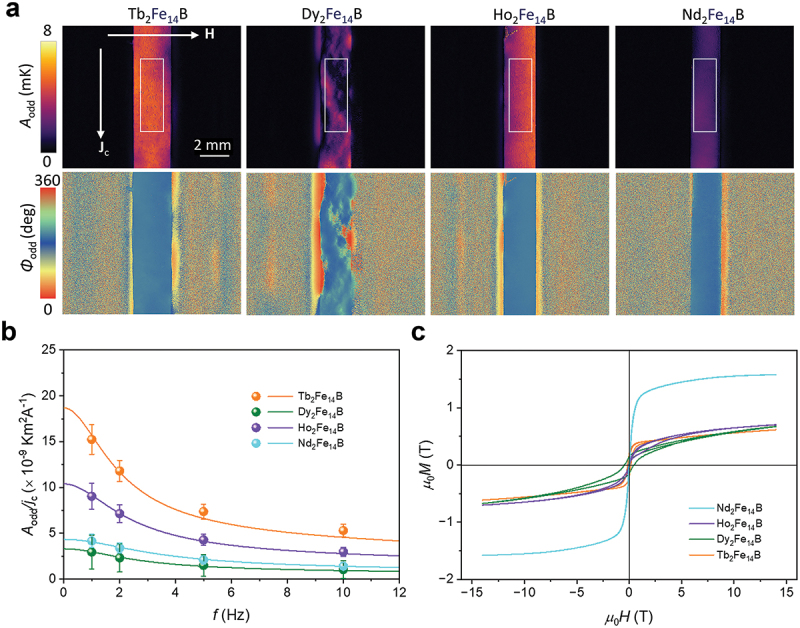
(a) *A*_odd_ and ϕ_odd_ images at *J*_c_ = 1.0 A, *µ*_0_*H* = ± 1 T, and *f* = 1 Hz for the RE_2_Fe_14_B (RE = Tb, Dy, Ho and Nd) alloys. (b) Frequency *f* dependence of *A*_odd_*/j*_c_. (c) Magnetic field *H* dependence of the magnetization *M* for the same RE_2_Fe_14_B (RE = Tb, Dy, Ho and Nd) alloys.

**Figure 4.**
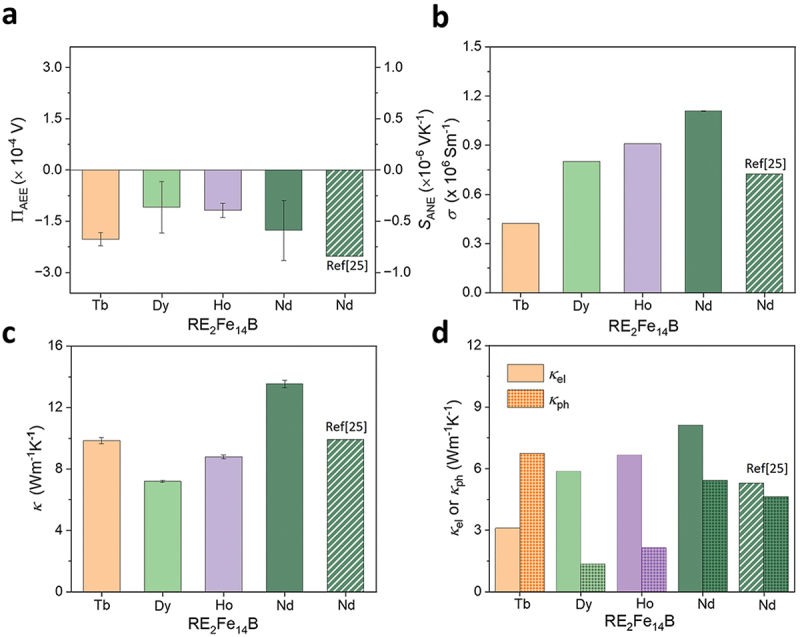
(a) $\Pi_{\mathrm{AEE}}$and *S*_ANE_ ($=\Pi_{\mathrm{AEE}}$/*T*), (b) *σ*, (c) *κ*, and (d) *κ*_el_ or *κ*_ph_ for the RE_2_Fe_14_B (RE = Tb, Dy, Ho and Nd) alloys. The error bars represent the standard deviation of the measurements.

Figure 3(a) represents the *A*_odd_ and ϕ_odd_ images at *J*_c_ = 1.0 A, *µ*_0_*H* = ± 1 T, and *f* = 1 hz for the RE_2_Fe_14_B slabs. A clear current-induced temperature modulation was observed on the surface of the slabs due to the generation of $j_{q,\mathrm{AEE}}$ along the *z* direction through the *x*-directional input **j**_c_ and *y*-directional **m**. Although the Tb_2_Fe_14_B, Ho_2_Fe_14_B, and Nd_2_Fe_14_B slabs show the uniform distribution of the *A*_odd_ and ϕ_odd_ signals, the inhomogeneous distribution of the signals was observed in Dy_2_Fe_14_B, which can be explained by that only a part of magnetic domains reacts to the applied magnetic field due to the largest magnetocrystalline anisotropy among the RE_2_Fe_14_B slabs (Figure 3(c)). Importantly, the sign of the AEE-induced temperature modulation on the surface for all the RE_2_Fe_14_B slabs is negative (ϕ_odd_ ~180${\;}^{\circ }$), suggesting the negative sign of Π_AEE_ and *S*_ANE_ [25]. This result indicates that the substitution of the RE site has a minor influence on the sign of Π_AEE_ and *S*_ANE_ for RE_2_Fe_14_B. To estimate the *S*_ANE_ value from the results of the LIT measurements, we measured the *f* dependence of *A*_odd_ per unit charge current density *j*_c_ (i.e. *A*_odd_*/j*_c_) for RE_2_Fe_14_B. The *A*_odd_ value at each *f* was defined by averaging the *A*_odd_ values within the area defined by the rectangle of 1.2 × 3.9 mm^2^ as indicated in Figure 3(a). As shown in Figure 3(b), the magnitude of *A*_odd_*/j*_c_ gradually decreases with increasing *f*, which can be well reproduced by considering the thermal diffusion in the sample via the one-dimensional heat diffusion equation in the frequency domain (solid curves in Figure 3(b)) [25,27]. From the fitting curve in Figure 3(b), we estimated the *A*_odd_*/j*_c_ value at the steady state, i.e. *f* = 0 hz, denoted as *A*_odd_^S^*/j*_c_ at 1 T. The magnetization *M* curves of RE_2_Fe_14_B were plotted in Figure 3(b). We found that *M* did not saturate at 1 T but increases up to 14 T (the maximum applied magnetic field in PPMS) (Figure 3(c)). Here, the *M* values at 14 T, *M*_14T_, are regarded as the saturation magnetization *M*_s_ because they are comparable to the reported *M*_s_ in the literature (Supplementary Table S1) [35]. $\Pi_{\mathrm{AEE}}$ at the saturation state is thus estimated as [26]

(3) $$\Pi_{\mathrm{AEE}}=\frac{2\kappa A_{odd}^{S}}{j_{c}t}\cdot \frac{M_{14T}}{M_{1T}}$$

where *t* is the thickness in the *z* direction. Figure 4(a) shows the Π_AEE_ and *S*_ANE_ values for the RE_2_Fe_14_B alloys. The calculated value of Π_AEE_ (*S*_ANE_) for Tb_2_Fe_14_B is −2.02 × 10^−4^ V (−0.67 × 10^−6^ VK^−1^), which is the highest among them and comparable to the commercial Nd_2_Fe_14_B permanent magnet [4,25]. It is noteworthy that the current RE_2_Fe_14_B alloys do not have any microstructure control and texture, leaving room for further improvement of *S*_ANE_ through the nano- and/or microstructure engineering [30], as suggested by the difference in *S*_ANE_ between our Nd_2_Fe_14_B and commercial Nd_2_Fe_14_B slabs. Figure 4(b) shows the *σ* values, gradually increased from 0.42 to 1.10 × 10^6^ Sm^−1^ by changing Tb to Nd in the RE_2_Fe_14_B systems. These findings show a similar trend with the work of Stankiewicz et al. which showed that heavy RE such as Tb_2_Fe_14_B systems exhibit lower electrical conductivity (1/*ρ*_*xx*_) than Nd_2_Fe_14_B system at ambient temperature [36]. Meanwhile, the commercial Nd_2_Fe_14_B-type permanent magnet shows 35% lower *σ* than the Nd_2_Fe_14_B ingot. Since the commercial Nd_2_Fe_14_B magnet had microstructural engineering, the charge carriers with low kinetic energies may get stopped by the presence of high density of grain boundaries, which leads to lower *σ* values compared to the ingots [37]. The *κ* values followed a similar trend to *σ* values in RE_2_Fe_14_B systems, except for Tb_2_Fe_14_B (Figure 4(c)). For a systematic understanding of *κ*, we estimated the electronic contribution $\kappa_{\mathrm{el}}$ and phonon contribution $\kappa_{\mathrm{ph}}$ of the thermal conductivity using $\kappa_{\mathrm{el}}=\mathrm{\sigma LT}$ and $\kappa_{\mathrm{ph}}=\kappa -\kappa_{\mathrm{el}}$, where *L* is the Lorenz number (2.44×10^−8^ WΩK^−2^) and *T* is the absolute temperature (300 K) [2,30]. Not only $\kappa_{\mathrm{el}}$ but also $\kappa_{\mathrm{ph}}$ shows a similar trend to *σ* except for Tb_2_Fe_14_B (Figure 4(d)). The uneven distribution of RE-rich phases (Figure 2(b)) may strongly scatter phonons at their interfaces with matrix, which is absent in the Tb₂Fe₁₄B, resulting in higher $\kappa_{\mathrm{ph}}\left[2\right]$. Interestingly, the commercial Nd_2_Fe_14_B-type permanent magnet shows lower *κ* values than our Nd_2_Fe_14_B ingot. The $\kappa_{\mathrm{el}}$ ($\kappa_{\mathrm{ph}}$) is decreased from 8.31 (5.22) Wm^−1^K^−1^ to 5.42 (4.49) Wm^−1^K^−1^ for the commercial Nd_2_Fe_14_B-type permanent magnet. The $\kappa_{\mathrm{el}}$ is decreased due to their low *σ* values and $\kappa_{\mathrm{ph}}$ is decreased due to microstructural engineering and significant reduction in grain size compared to current ingots, which may induce the phonon scattering [37]. As a result, the obtained total *κ* values are lower than our ingot. Nevertheless, the intergranular phase particularly in the ultra-fine grain sized permanent magnets influences the thermal conductivity, as demonstrated by Kautsar et al. in the grain boundary engineered hot-deformed Nd-Fe-B magnets [38]

### 3.3. Evaluation of contribution factors for ANE in RE_2_Fe_14_B alloys

**Figure 5.**
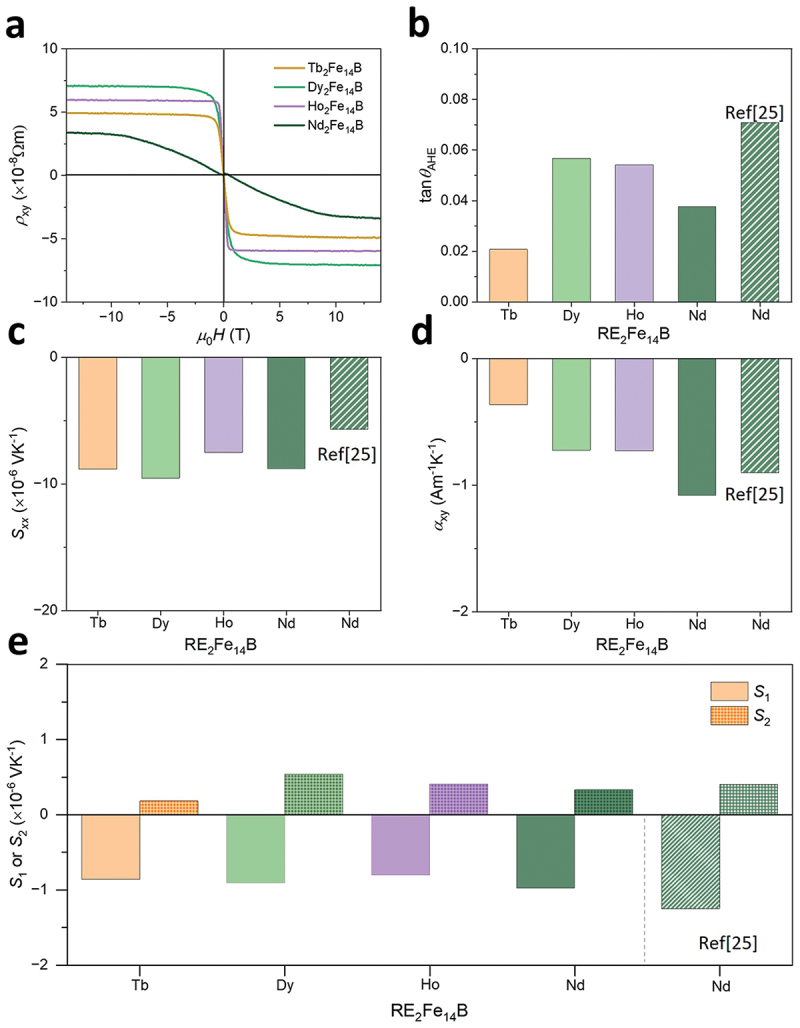
(a) *ρ*_*xy*_, (b) tanθ_AHE_, (c) *S*_*xx*_, (d) *α*_*xy*_, and (e) *S*_1_ and *S*_2_ for the RE_2_Fe_14_B (RE = Tb, Dy, Ho and Nd) alloys.

The dominant contribution in ANE/AEE for our RE_2_Fe_14_B systems is distinguished as follows. *S*_ANE_ can be divided into two components as [7,10,12–15,27,39]:

(4) $$S_{\mathrm{ANE}}=\rho_{\mathrm{xx}}\alpha_{\mathrm{xy}}+\rho_{\mathrm{xy}}\alpha_{\mathrm{xx}}=S_{1}+S_{2}$$

where $\rho_{\mathrm{xx}}=1/\sigma$ is the longitudinal resistivity and $\alpha_{\mathrm{xx}}\left(\alpha_{\mathrm{xy}}\right)$ the diagonal component (off-diagonal component) of the thermoelectric conductivity tensor. *S*_1_ originates from the transverse thermoelectric conductivity ($\alpha_{\mathrm{xy}}$) and *S*_2_ is attributed to the synergistic action of the Seebeck effect and AHE. The *S*_2_ term can be written as *S*_2_ = –*S*_*xx*_tan*θ*_AHE_, where *S*_*xx*_ = $\rho_{\mathrm{xx}}\alpha_{\mathrm{xx}}$ and tan*θ*_AHE,_ = $-\rho_{\mathrm{xy}}/\rho_{\mathrm{xx}}$ with *θ*_AHE_ being the anomalous Hall angle [25,30]. We first evaluated tan*θ*_AHE_ by measuring the *H* dependence of $\rho_{\mathrm{xy}}$ of the samples. Figure 5(a) shows $\rho_{\mathrm{xy}}$ as a function of *H* for the RE_2_Fe_14_B samples. In the present work, we assumed that the contributions of the ordinary Nernst/Ettingshausen effect and the magnetic field dependence of the longitudinal transport properties are negligibly small in our samples. Using the $\rho_{\mathrm{xy}}$ values at *µ*_0_*H* = 14 T, taken as the AHE contribution, we calculated the tan*θ*_AHE_ values as shown in Figure 5(b). The tan*θ*_AHE_ values are positive for all the RE_2_Fe_14_B samples and Dy_2_Fe_14_B shows the highest tan*θ*_AHE_. Figure 5(c) represents *S*_*xx*_ for the RE_2_Fe_14_B samples. The magnitude of *S*_*xx*_ is comparable for all the RE_2_Fe_14_B samples. Figure 5(d) shows $\alpha_{\mathrm{xy}}$, estimated by substituting *S*_ANE_, $\rho_{\mathrm{xx}}$, *S*_*xx*_, and tan*θ*_AHE_ into Equation (4). The RE_2_Fe_14_B samples show negative $\alpha_{\mathrm{xy}}$ and Nd_2_Fe_14_B shows the highest value among them. Furthermore, the *S*_1_ and *S*_2_ contributions to AEE/ANE are shown in Figure 5(e). *S*_ANE_ is determined by the compensation of the *S*_1_ and *S*_2_ contributions. It is clearly observed that the suppression of *S*_2_ term while sustaining large negative *S*_1_ term majorly decides the magnitude of the *S*_ANE_ values in the RE_2_Fe_14_B samples.

### 3.4. Structural analysis of Nd_2_(Fe_1-p_Co_p_)_14_B alloys

**Figure 6.**
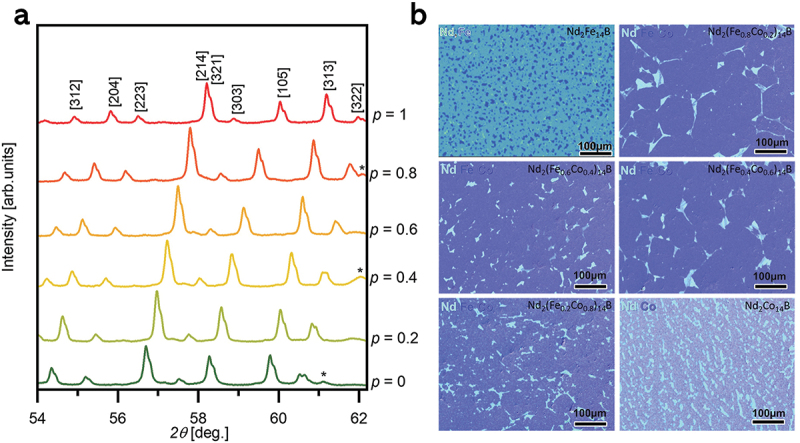
(a) Crystal structure analysis using XRD and (b) chemical composition analysis using SEM-EDS for the Nd_2_(Fe_1-*p*_Co_*p*_)_14_B (*p* = 0, 0.2, 0.4, 0.6, 0.8, and 1.0) alloys.

In this section, we did the same investigation on the substitution of the Fe site with Co in the Nd_2_(Fe_1*-p*_Co_*p*_)_14_B alloys. The phase and purity of the Nd_2_(Fe_1-*p*_Co_*p*_)_14_B (*p* = 0, 0.2, 0.4, 0.6, 0.8, and 1.0) homogenized alloys are depicted in Figure 6(a,b), respectively. XRD peak positions shift towards higher *2θ* angles upon the Co substitution in Fe site in the Nd_2_(Fe_1*-p*_Co_*p*_)_14_B (*p* = 0, 0.2, 0.4, 0.6, 0.8, and 1.0) alloys, evidencing a decrease in the lattice parameters. Rietveld refinement of the XRD patterns confirms the decrease in the lattice parameters *a* and *c* with the substitution of Fe for Co, by a total of 1.78% and 2.75%, respectively (see Table 1). This decrease in the lattice parameters is in agreement with the smaller atomic radius of Co compared to that of Fe, as well as previous literature [40]. The Rietveld refinement analysis of XRD (Supplementary Figures S5–S9) revealed that in addition to 2:14:1 matrix phase, some minor phases which often form as metallic or oxide phases in the 2:14:1 system including bcc-Fe, NdCo_2_, NdCo_4_B, and NdCo_5_ exist in the homogenized Nd_2_(Fe_1*-p*_Co_*p*_)_14_B alloys [41]. However, precise identification of the few secondary phases remains challenging, as they are present in a small volume fraction and might not be able to detectable in low-magnification SEM images, which provide more local information.

**Table 1.** Lattice parameters for Nd_2_(Fe_*1-p*_Co_*p*_)_14_B (*p* = 0, 0.2, 0.4, 0.6, 0.8, and 1) alloys.

Nd_2_(Fe_1*-p*_Co_*p*_)_14_B	*a (Å)*	*c (Å)*
*p* = 0	8.808	12.208
*p* = 0.2	8.786	12.162
*p* = 0.4	8.749	12.112
*p* = 0.6	8.711	12.051
*p* = 0.8	8.676	11.972
*p* = 1.0	8.651	11.872

### 3.5. Transverse thermoelectric properties of Nd_2_(Fe_1-x_Co_x_)_14_B alloys

**Figure 7.**
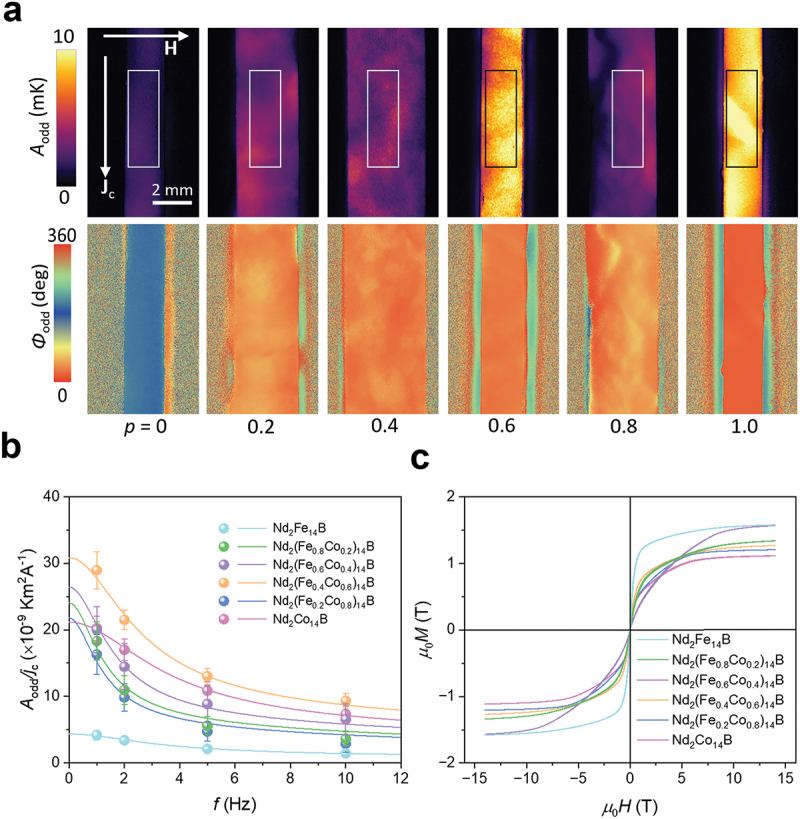
(a) *A*_odd_ and ϕ_odd_ images at *J*_c_ = 1.0 A, *µ*_0_*H* = ± 1 T, and *f* = 1 Hz for the Nd_2_(Fe_1-*p*_Co_*p*_)_14_B (*p* = 0, 0.2, 0.4, 0.6, 0.8, and 1.0) alloys. (b) *f* dependence of *A*_odd_*/j*_c_. (c) *H* dependence of *M* for the same Nd_2_(Fe_1-*p*_Co_*p*_)_14_B (*p* = 0, 0.2, 0.4, 0.6, 0.8, and 1.0) alloys.

The LIT technique was utilized to investigate AEE in the Nd_2_(Fe_1*-p*_Co_*p*_)_14_B samples in the same manner as the previous experiments. The extracted *A*_odd_ and ϕ_odd_ images for Nd_2_(Fe_1*-p*_Co_*p*_)_14_B are shown in Figure 7(a). The clear current-induced temperature modulation was obtained on the whole surface of the slabs. Although the ϕ_odd_ image of Nd_2_Fe_14_B shows ~180°, the Co-substituted Nd_2_(Fe_1*-p*_Co_*p*_)_14_B samples show a phase reversal to ~0° on the top surface [25,27,30]. The substitution of the transition metal in Nd_2_Fe_14_B plays a significant role on the sign of Π_AEE_ and *S*_ANE_. Figure 7(b) shows the *f* dependence of the *A*_odd_/*j*_c_ values for the Nd_2_(Fe_1*-p*_Co_*p*_)_14_B slabs, which are estimated by averaging the *A*_odd_ values within the area defined by the rectangle of 1.5 × 4.9 mm^2^ as indicated in Figure 7(a). The Co-substituted Nd_2_(Fe_1*-p*_Co_*p*_)_14_B samples show higher magnitude of *A*_odd_*/j*_c_ than the pristine Nd_2_Fe_14_B and Nd_2_(Fe_0.4_Co_0.6_)_14_B exhibits the highest *A*_odd_^S^*/j*_c_ value among them. The magnetization curves for the Nd_2_(Fe_1*-p*_Co_*p*_)_14_B alloys are plotted in Figure 7(c).

**Figure 8.**
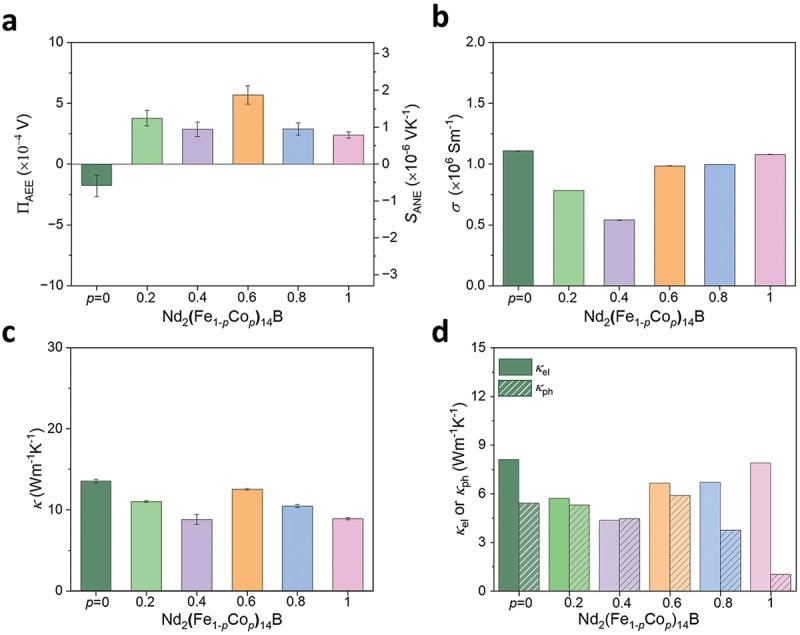
(a) $\Pi_{\mathrm{AEE}}$and *S*_ANE_ ($=\Pi_{\mathrm{AEE}}$/*T*), (b) *σ*, (c) *κ*, and (d) *κ*_el_ or *κ*_ph_ for the Nd_2_(Fe_*1-p*_Co_*p*_)_14_B (*p* = 0, 0.2, 0.4, 0.6, 0.8, and 1.0) alloys. The error bars represent the standard deviation of the measurements.

Figure 8(a) shows the Π_AEE_ and *S*_ANE_ values for the Nd_2_(Fe_1*-p*_Co_*p*_)_14_B alloys. The Π_AEE_ (*S*_ANE_) value significantly improves more than twice in Nd_2_(Fe_0.4_Co_0.6_)_14_B. Note that no strong correlation was found between the *S*_ANE_ and *M*_s_ (Supplementary Figure S10) [5]. The *σ* and *κ* values are shown in Figure 8(b,c), respectively. The magnitude of the *σ* values exhibits a minimum for *p* = 0.4 in the Nd_2_(Fe_1*-p*_Co_*p*_)_14_B alloys. Gu et al. reported that Fe *d*-orbital electrons primarily contribute to the total density of state (TDOS) at the Fermi level, with 2.31 states/eV atom in Nd₂Fe₁₄B. This value is decreased to 2.06 states/eV atom when Co replaced for Fe, where the Co *d*-orbital electrons become responsible for the TDOS at Fermi level in Nd₂Co₁₄B [42,43]. By assuming the rigid band model, the decrease in *σ* observed in the present work can be attributed to the slight reduction in TDOS at the Fermi level due to domination of the Co *d*-orbital electrons in the Nd_2_(Fe_1*-p*_Co_*p*_)_14_B system. On the other hand, the *κ* values are slightly decreased for the Co-substituted Nd_2_(Fe_1*-p*_Co_*p*_)_14_B alloys, and the values are spread in the 9–12 Wm^−1^K^−1^ range. The $\kappa_{\mathrm{el}}$and $\kappa_{\mathrm{ph}}$ contributions to the total *κ* are shown in Figure 8(d). The $\kappa_{\mathrm{ph}}$ has similar values but is significantly reduced for Nd_2_Co_14_B. The alloys with *p* ≥0.8 have a higher density of Nd-rich phases (Figure 6(b)) compared to the others, which may scatter the phonons at their interfaces with matrix, leading to a decrease in $\kappa_{\mathrm{ph}}\left[2\right]$.

### 3.6. Evaluation of contribution factors for ANE in Nd_2_(Fe_1-p_Co_p_)_14_B alloys

**Figure 9.**
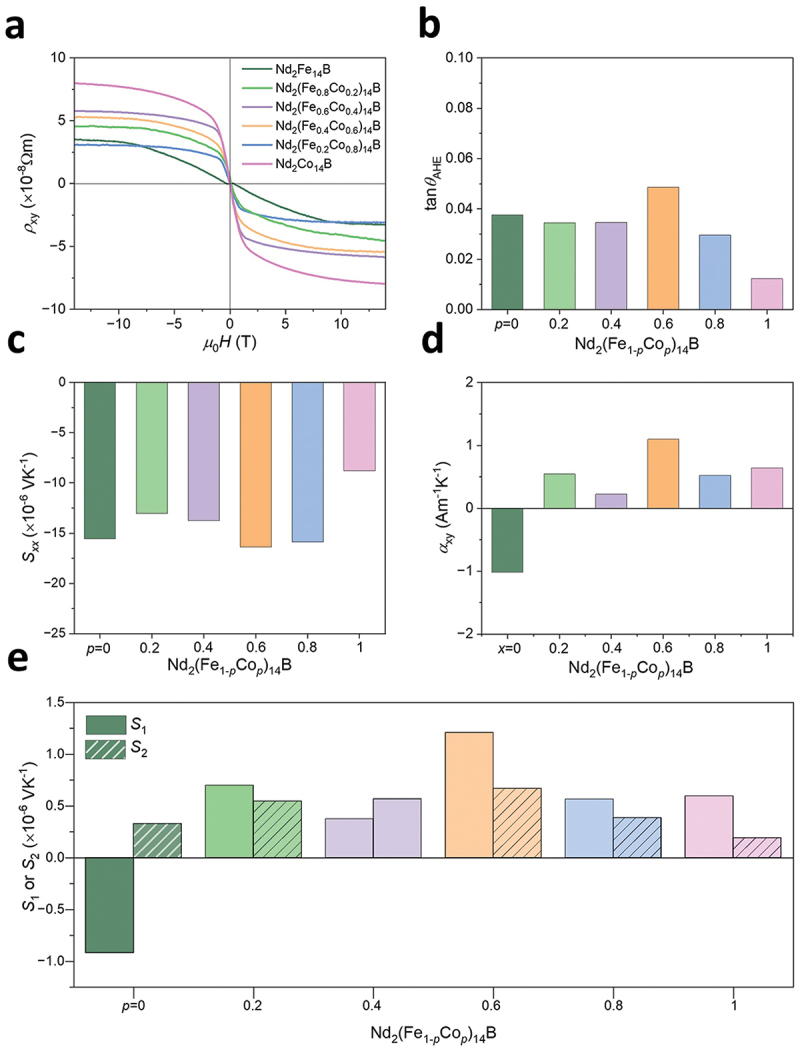
(a) *ρ*_*xy*_, (b) tanθ_AHE_, (c) *S*_*xx*_, (d) *α*_*xy*_, and (e) *S*_1_ and *S*_2_ for the Nd_2_(Fe_*1-p*_Co_*p*_)_14_B (*p* = 0, 0.2, 0.4, 0.6, 0.8, and 1.0) alloys.

To elucidate the contribution factors for transverse thermoelectric properties in the Nd_2_(Fe_1-*p*_Co_*p*_)_14_B alloys, we estimated *S*_1_ and *S*_2_. The *H* dependence of $\rho_{\mathrm{xy}}$ for the Nd_2_(Fe_1-*p*_Co_*p*_)_14_B alloys is shown in Figure 9(a). The $\rho_{\mathrm{xy}}$ values at *µ*_0_*H* = 14 T are taken as the AHE contribution. By using the $\rho_{\mathrm{xy}}$ values, tan*θ*_AHE_ is evaluated and shown in Figure 9(b). The estimated tan*θ*_AHE_ values are positive for all the Nd_2_(Fe_1*-p*_Co_*p*_)_14_B samples, and Nd_2_Co_14_B shows the smallest magnitude among them. The *S*_*xx*_ values for all the ingots are shown in Figure 9(c) and all of them exhibit negative sign. The magnitude of *S*_*xx*_ is comparable for all samples except for Nd_2_Co_14_B. Using the obtained *S*_ANE_, *S*_*xx*_, and tan*θ*_AHE_ values, $\alpha_{\mathrm{xy}}$ is estimated and shown in Figure 9(d). It is found that, although the pristine Nd_2_Fe_14_B exhibits negative sign of $\alpha_{\mathrm{xy}}$, the Co-substituted Nd_2_(Fe_1*-p*_Co_*p*_)_14_B samples display positive sign. This change might be due to the modification of the TDOS at the Fermi level, primarily caused by the change in the lattice parameter and contribution of Co *d*-orbital electrons in the Co-rich Nd_2_Fe_14_B alloys [42,43]. Notably, the sign of $\alpha_{\mathrm{xy}}$ is prominently responsible for the sign changes in the *S*_ANE_ values of Nd_2_(Fe_1*-p*_Co_*p*_)_14_B. The obtained *S*_1_ and *S*_2_ values are positive for the Co-substituted Nd_2_(Fe_1-*p*_Co_*p*_)_14_B (Figure 9(e)). Based on the outcomes, it is argued that the *S*_1_ and *S*_2_ values constructively contribute to the total *S*_ANE._

**Figure 10.**
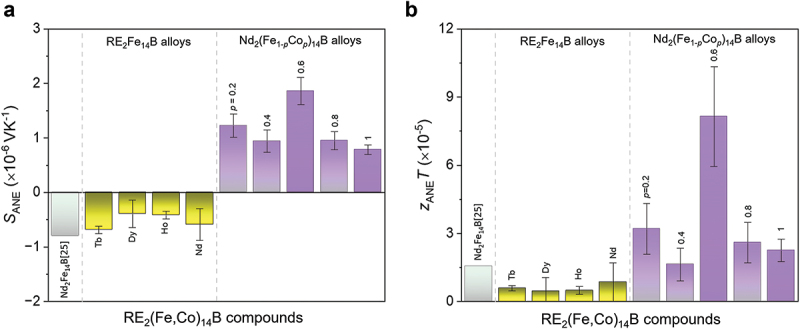
Comparison of (a) *S*_ANE_ and (b) $z_{\mathrm{ANE}}T$ for commercial Nd_2_Fe_14_B permanent magnets with those for our alloys measured at room temperature. The error bars represent the standard deviation of the measurements.

Finally, we quantitatively compared the *S*_ANE_ and dimensionless figure of merit $z_{\mathrm{ANE}}T$ values for the commercial Nd_2_Fe_14_B permanent magnet in the literature with our alloys (Figure 10(a,b)). $z_{\mathrm{ANE}}T$ for transverse thermoelectric conversion due to ANE is estimated by the following expression [25–27,30]:

(5) $$z_{\mathrm{ANE}}T=\frac{\Pi_{AEE}^{2}\sigma }{\kappa }\frac{1}{T}=\frac{S_{ANE}^{2}\sigma }{\kappa }T$$

Using the measured *S*_ANE_, *σ* and *κ* values, $z_{\mathrm{ANE}}T$ is estimated using Equation (5). Importantly, the Tb_2_Fe_14_B and Nd_2_Fe_14_B alloys show almost similar negative *S*_ANE_, but lower $z_{\mathrm{ANE}}T$ than the commercial Nd_2_Fe_14_B magnet due to higher *κ* values. In turn, the Nd_2_(Fe_1*-p*_Co_*p*_)_14_B alloys show the sign change and significant improvement in *S*_ANE_ as well as $z_{\mathrm{ANE}}T$. The largest estimated $z_{\mathrm{ANE}}T$ of 8.29 × 10^−5^ is obtained for the Nd_2_(Fe_0.4_Co_0.6_)_14_B composition due to their higher *S*_ANE_. We found that $z_{\mathrm{ANE}}T$ in the Nd_2_(Fe_0.4_Co_0.6_)_14_B alloys is more than nine folds higher than that of the pristine Nd_2_Fe_14_B alloy. These results highlight the importance of fundamental research to improve the transverse thermoelectric properties of compounds with large magnetocrystalline anisotropy. Such studies will guide material selection for the development of permanent magnets with optimized micro/nanostructures for transverse thermoelectric generation devices with improved anomalous Nernst power density.

## 4. Conclusions

We systematically investigated ANE/AEE in RE-based alloys with large magnetocrystalline anisotropy for ANE-based TEG devices operating without an external magnetic field. We observed that all RE_2_Fe_14_B alloys exhibit negative *S*_ANE_ due to the negative sign of the transverse thermoelectric conductivity $\alpha_{\mathrm{xy}}$ regardless of the RE element. Among them, Tb_2_Fe_14_B compound shows the largest negative *S*_ANE_, comparable to that of the commercially available Nd_2_Fe_14_B-type permanent magnet. Our work also confirmed that substituting Co for Fe site in the Nd_2_(Fe_1*-p*_Co_*p*_)_14_B compounds alters the sign of *S*_ANE_. A more than twofold improvement in the magnitude of *S*_ANE_ was obtained in the Nd_2_(Fe_0.4_Co_0.6_)_14_B alloy compared with the pristine Nd_2_Fe_14_B. Both *S*_1_ and *S*_2_ contributions constructively increase *S*_ANE_ in the Nd_2_(Fe_1-*p*_Co_*p*_)_14_B compounds. The *S*_ANE_ values of the developed RE-TM-B alloys are comparable to those of existing commercial permanent magnets. This result suggests a significant potential for further enhancement of *S*_ANE_ by controlling microstructures and processing parameters, paving the way for sustainable energy applications using ANE-based TEG devices.

## Supplementary Material

Supplemental Material

Supplemental Material
