# Supplementary material for: Systematic investigation on transverse thermoelectric conversion of RE2(Fe,Co)14B (RE = rare-earth) compounds
Source: Sci Technol Adv Mater. 2025 Jun 18;26(1):2520162. doi: 10.1080/14686996.2025.2520162 (PMC12261519; doi:10.1080/14686996.2025.2520162)
Supplement: Supplemental Material [file TSTA_A_2520162_SM4249.pdf]

Supplementary Information

Systematic investigation on transverse thermoelectric conversion of $RE_2(Fe,Co)_{14}B$ (RE = rare-earth) compounds

Babu Madavali[a*], Fuyuki Ando[a*†], Takamasa Hirai[a], Andres Martin-Cid[a], Ken-ichi Uchida[a,b], and Hossein Sepehri-Amin[a†]

[a]Research Center for Magnetic and Spintronic Materials, National Institute for Materials Science, Tsukuba, Japan; [b]Department of Advanced Materials Science, Graduate School of Frontier Sciences, The University of Tokyo, Kashiwa, Japan

[*]These authors contributed equally to this work.

[†]Corresponding authors: ANDO.Fuyuki@nims.go.jp, H.SEPEHRIAMIN@nims.go.jp

Composition	$\mu_0 M_s$ (T)	$\mu_0 M_s$ (T) [32]
$Tb_2Fe_{14}B$	0.61	0.70
$Dy_2Fe_{14}B$	0.67	0.71
$Ho_2Fe_{14}B$	0.70	0.81
$Nd_2Fe_{14}B$	1.57	1.60

Supplementary Table 1. Comparison of saturation magnetization $\mu_0 M_s$ (T) for $RE_2Fe_{14}B$ ferromagnets [ref 35].

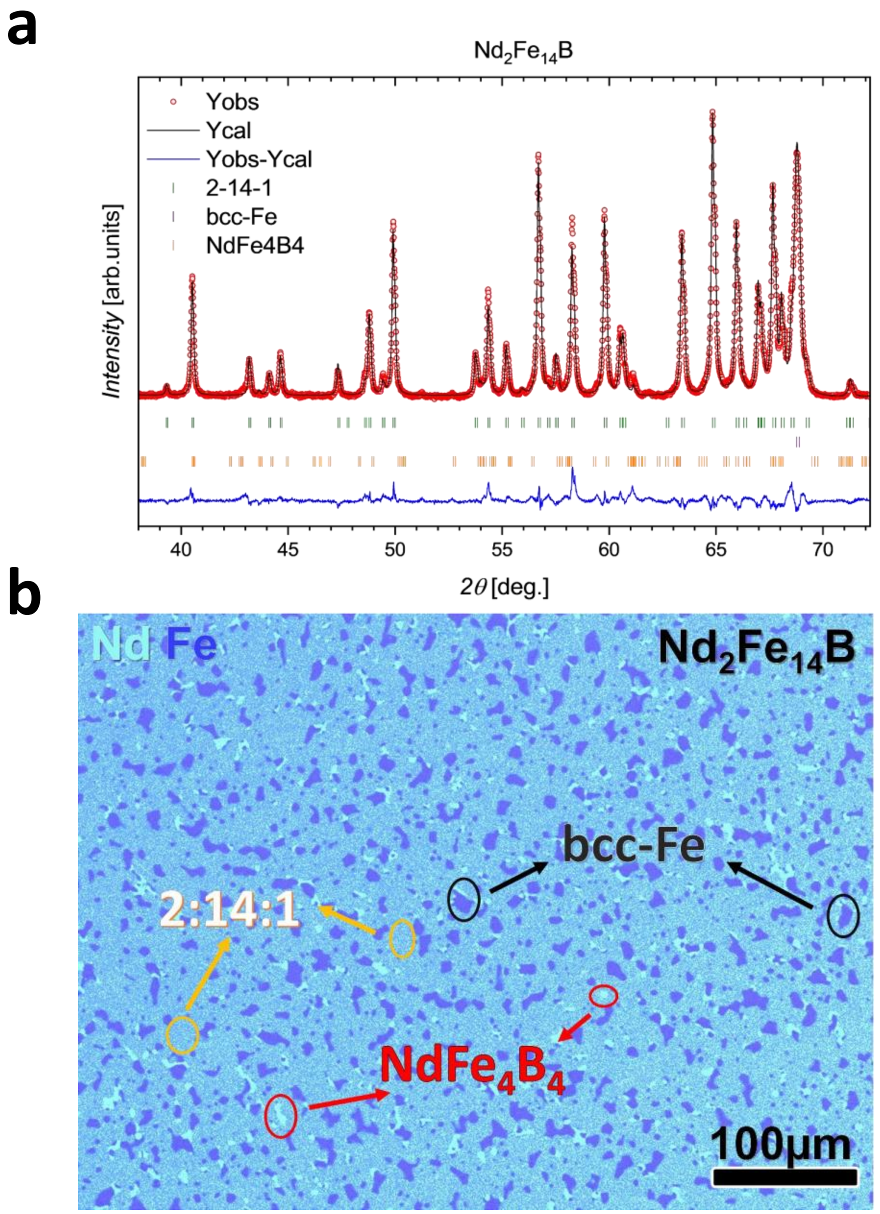

Supplementary Figure S1: (a) Rietveld analysis and (b) chemical composition analysis using SEM-EDS for the $Nd_2Fe_{14}B$ alloys

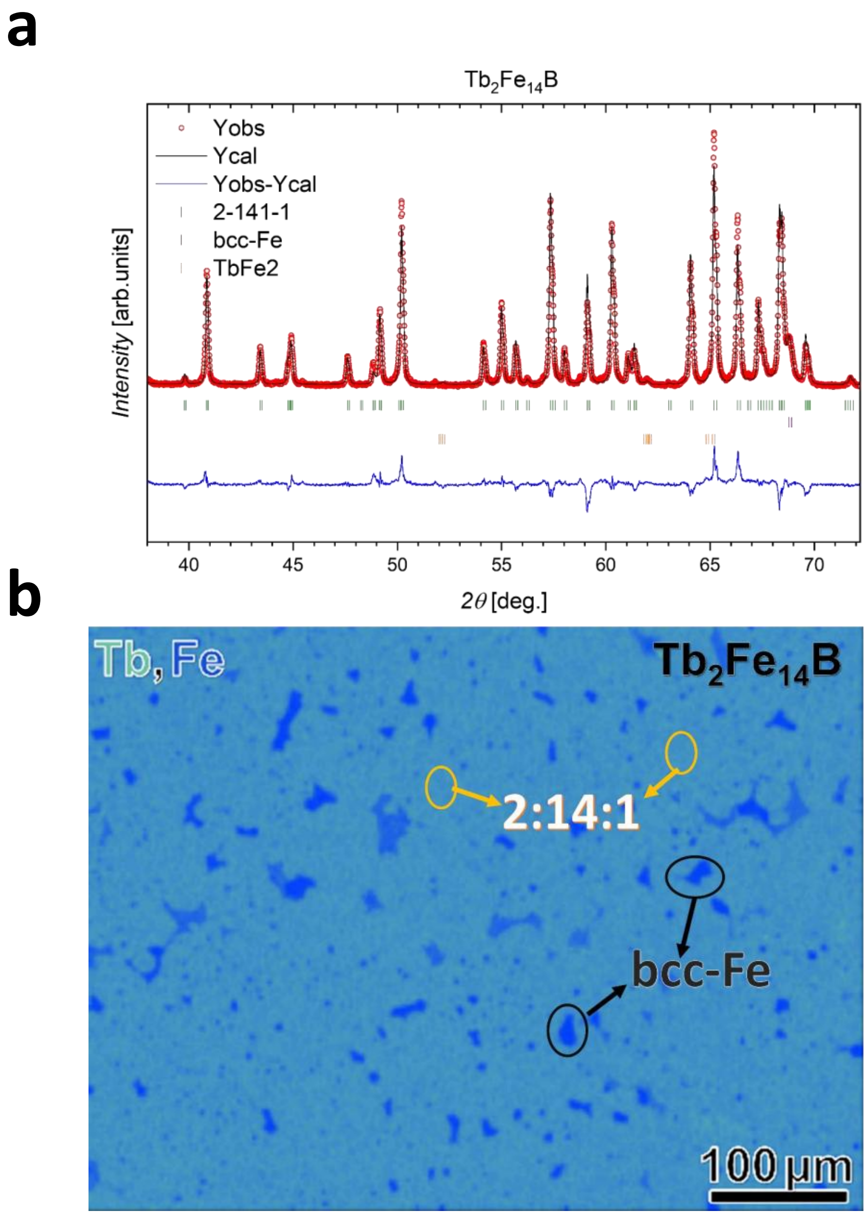

Supplementary Figure S2: (a) Rietveld analysis and (b) chemical composition analysis using SEM-EDS for the $Tb_2Fe_{14}B$ alloys

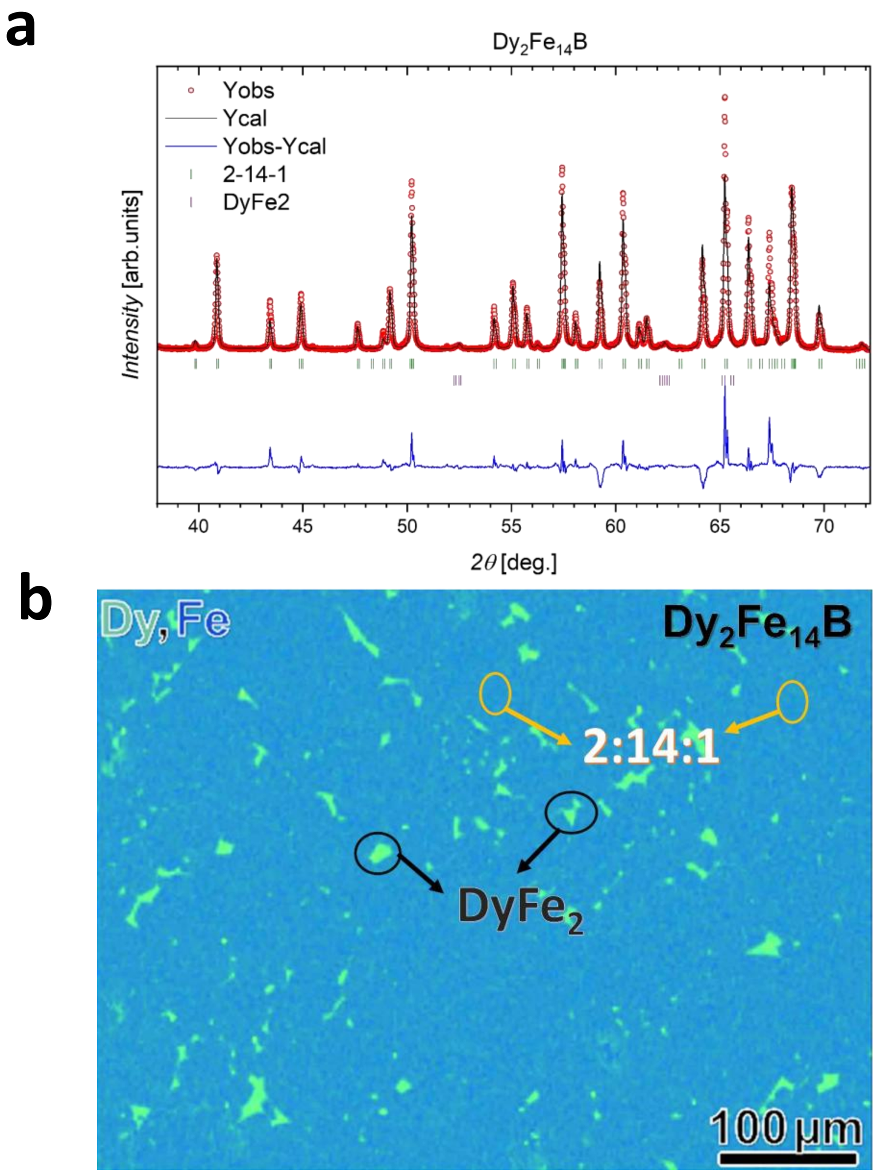

Supplementary Figure S3: (a) Rietveld analysis and (b) chemical composition analysis using SEM-EDS for the $Dy_2Fe_{14}B$ alloys

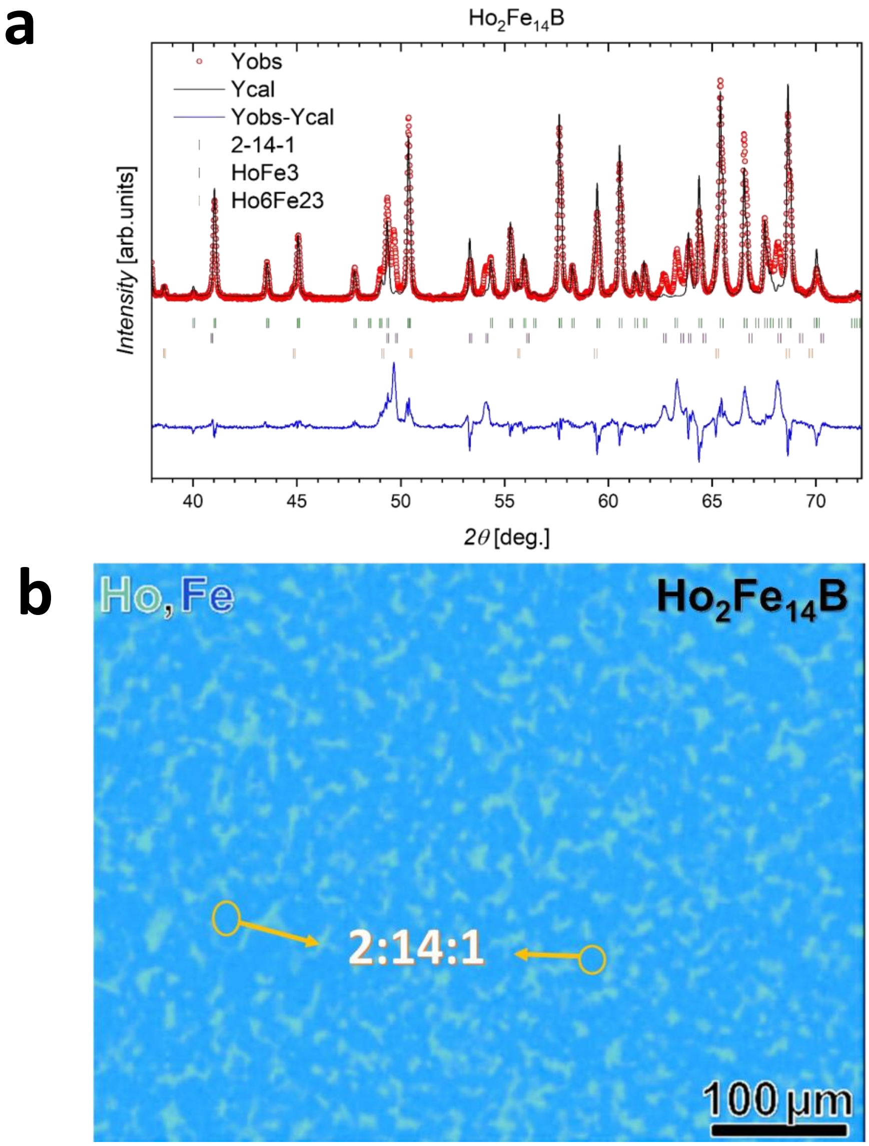

Supplementary Figure S4: (a) Rietveld analysis and (b) chemical composition analysis using SEM-EDS for the $Ho_2Fe_{14}B$ alloys. The identification of all secondary phases in the $Ho_2Fe_{14}B$ system remains challenging in SEM-EDS, and some of the secondary reflections found in XRD would not be identified with the existing phases.

a

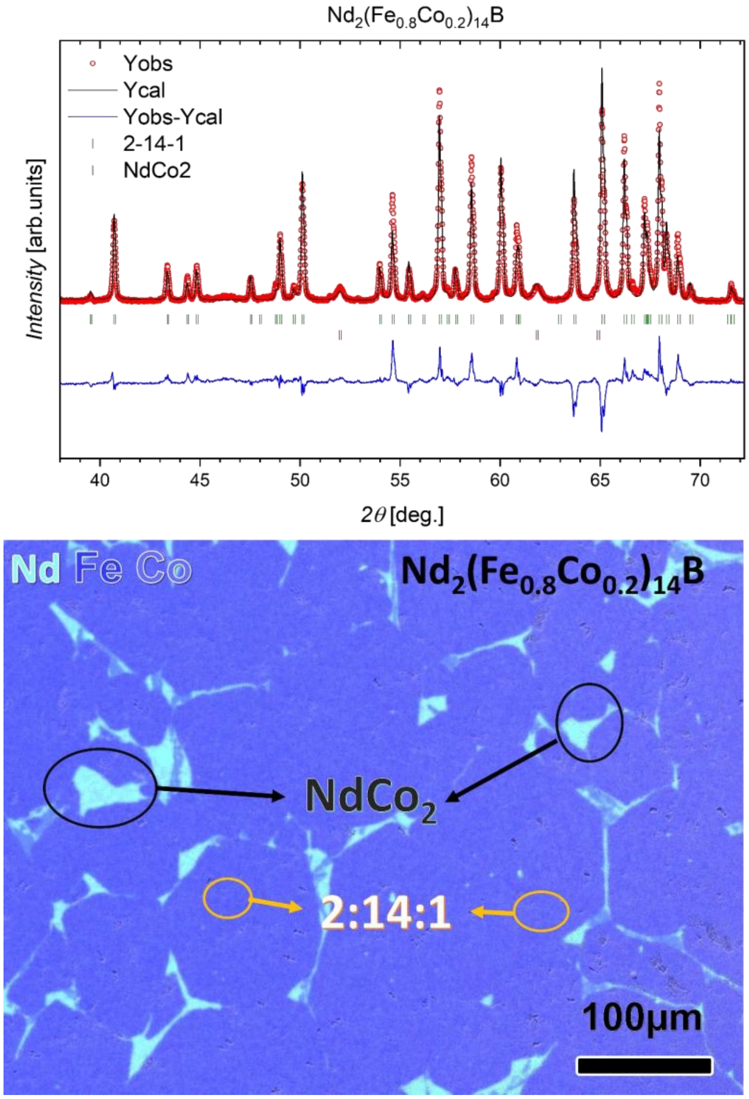

b

Supplementary Figure S5: (a) Rietveld analysis and (b) chemical composition analysis using SEM-EDS for the $Nd_2(Fe_{0.8}Co_{0.2})_{14}B$ alloys

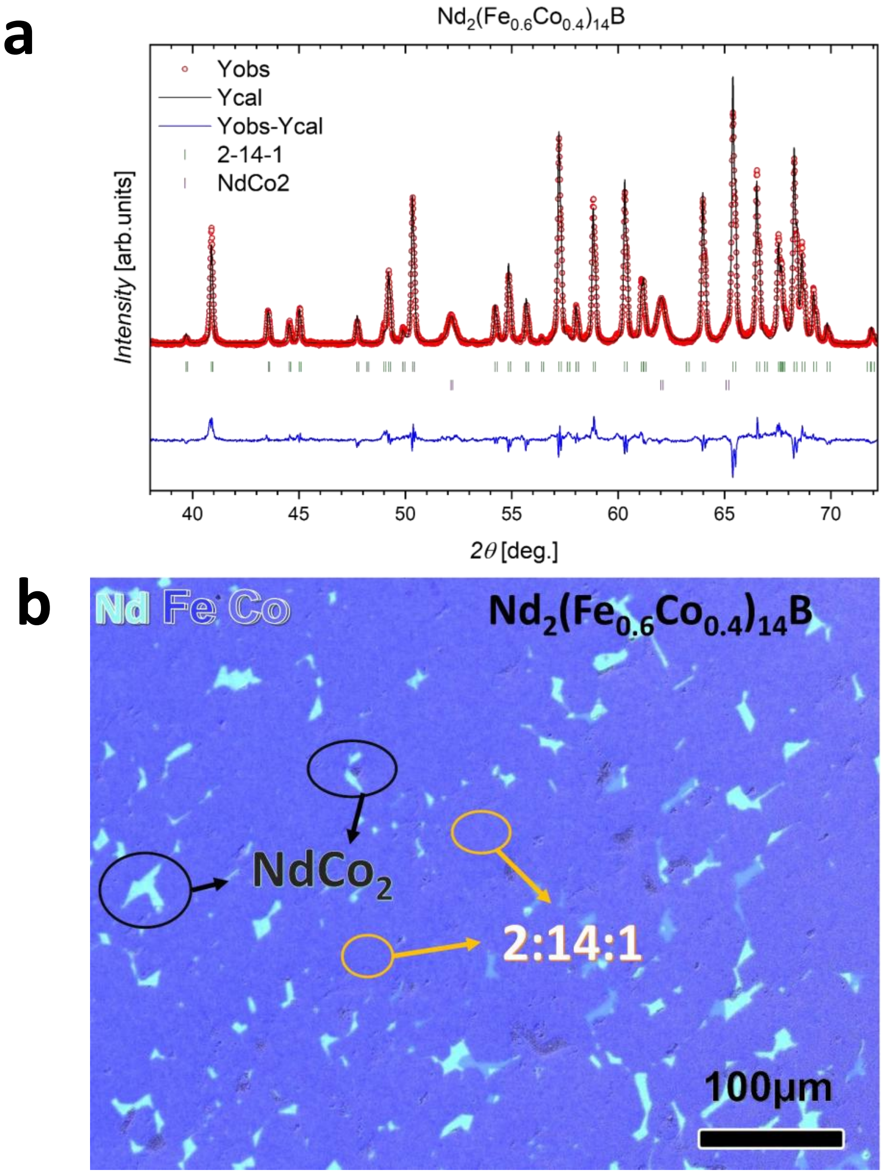

Supplementary Figure S6: (a) Rietveld analysis and (b) chemical composition analysis using SEM-EDS for the $Nd_2(Fe_{0.6}Co_{0.4})_{14}B$ alloys

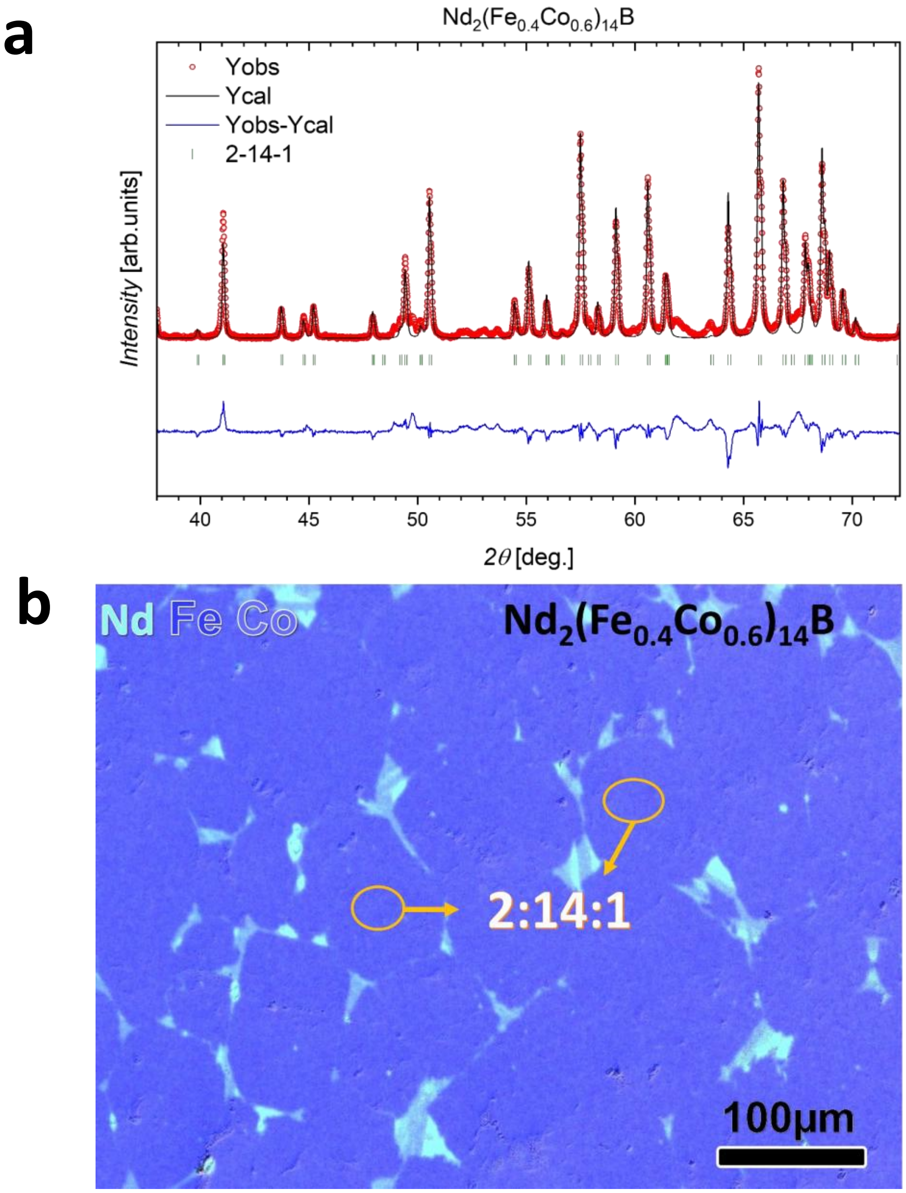

Supplementary Figure S7: (a) Rietveld analysis and (b) chemical composition analysis using SEM-EDS for the $Nd_2(Fe_{0.4}Co_{0.6})_{14}B$ alloys. The secondary phases at all triple junctions contain both RE-rich and RE-deficiency regions observed in the SEM-EDS image. Precise identification of these secondary phases remains challenging, as they are present in small volume fraction and might not be able to detectable in low-magnification SEM images, which provide more local information.

a

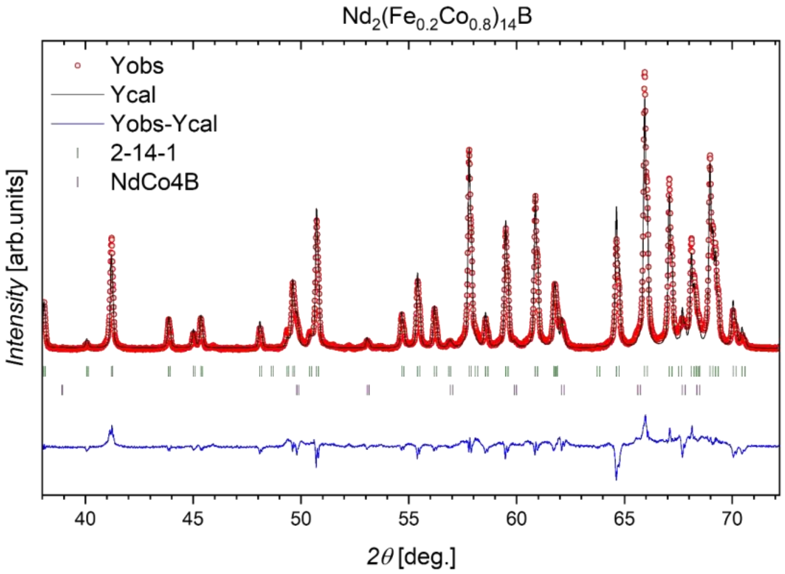

b

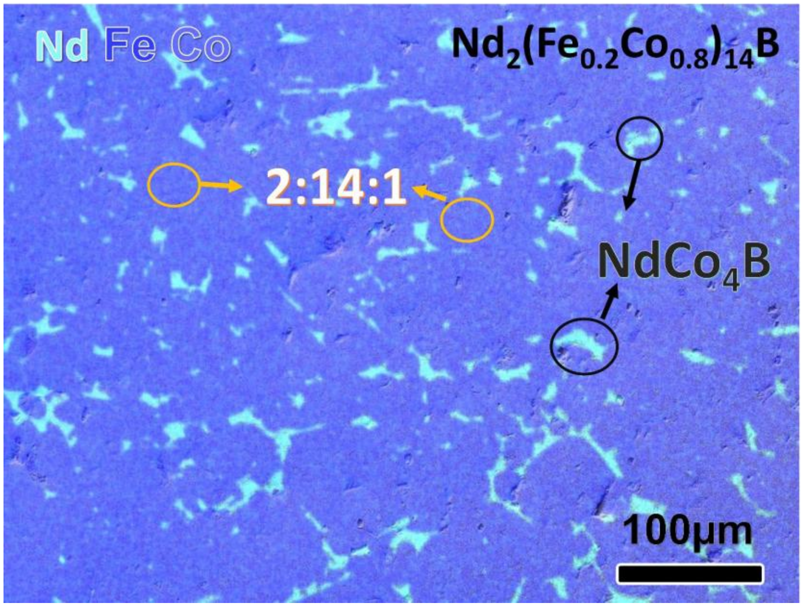

Supplementary Figure S8: (a) Rietveld analysis and (b) chemical composition analysis using SEM-EDS for the $Nd_2(Fe_{0.2}Co_{0.8})_{14}B$ alloys

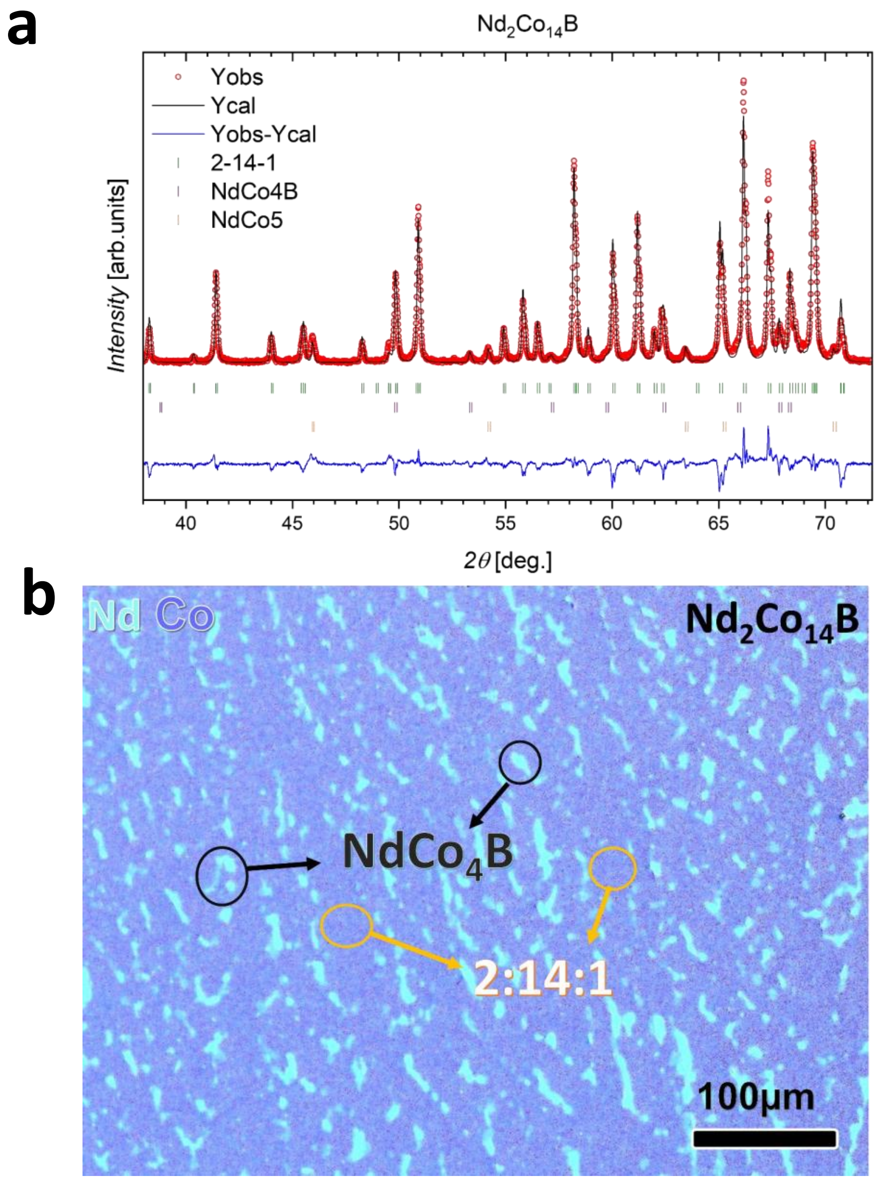

Supplementary Figure S9: (a) Rietveld analysis and (b) chemical composition analysis using SEM-EDS for the $Nd_2Co_{14}B$ alloys

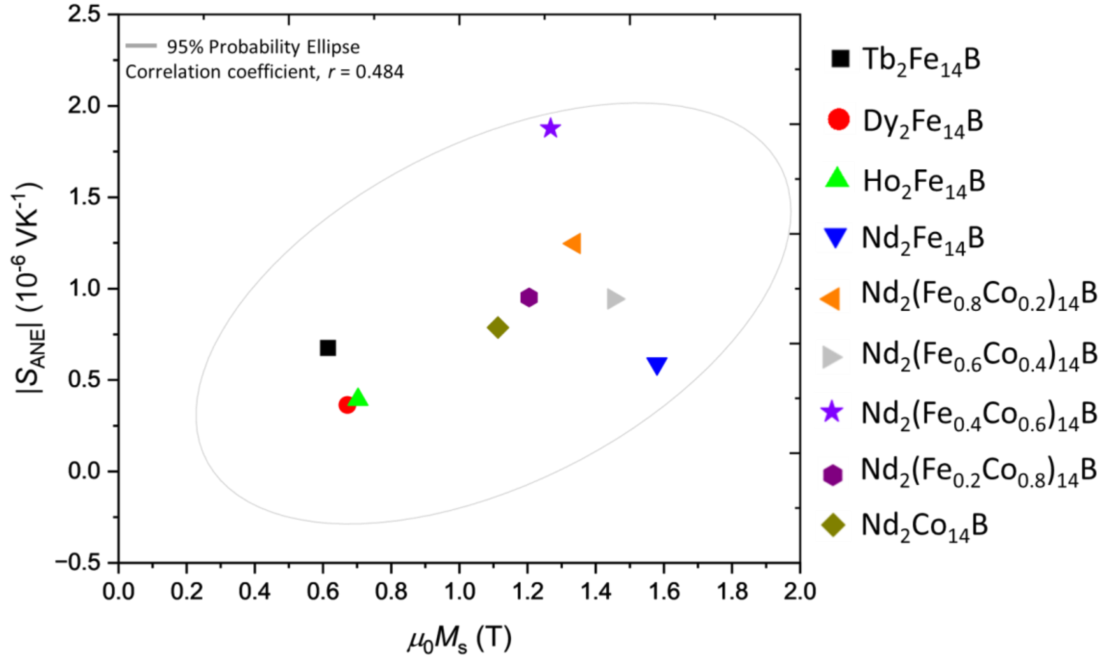

Supplementary Figure S10: Saturation magnetization M_s dependence of the absolute values of the anomalous Nernst coefficient $|S_{ANE}|$ for $RE_2(Fe,Co)_{14}B$ (RE = rare-earth) alloys. The estimated correlation coefficient, r = 0.484 indicated the weak to moderate positive linear correlation between M_s and ANE coefficient in our work.
