# Supplementary material for: Systematic investigation on transverse thermoelectric conversion of RE2(Fe,Co)14B (RE = rare-earth) compounds
Source: Sci Technol Adv Mater. 2025 Jun 18;26(1):2520162. doi: 10.1080/14686996.2025.2520162 (PMC12261519; doi:10.1080/14686996.2025.2520162)
Supplement: Supplemental Material [file TSTA_A_2520162_SM3314.docx]

**Supplementary Information**

**Systematic investigation on transverse thermoelectric conversion of**

**RE_2_(Fe,Co)_14_B (RE = rare-earth) compounds**

Babu Madavali^a*^, Fuyuki Ando^a*†^, Takamasa Hirai^a^, Andres Martin-Cid^a^,

Ken-ichi Uchida^a,b^, and Hossein Sepehri-Amin^a†^

*^a^Research Center for Magnetic and Spintronic Materials, National Institute for Materials Science, Tsukuba, Japan; ^b^Department of Advanced Materials Science, Graduate School of Frontier Sciences, The University of Tokyo, Kashiwa, Japan*

^*^These authors contributed equally to this work.

**^†^**Corresponding authors: ANDO.Fuyuki@nims.go.jp, H.SEPEHRIAMIN@nims.go.jp

| Composition | *μ*_0_*M*_s_ (T) | *μ*_0_*M*_s_ (T) [32] |
| --- | --- | --- |
| Tb_2_Fe_14_B | 0.61 | 0.70 |
| Dy_2_Fe_14_B | 0.67 | 0.71 |
| Ho_2_Fe_14_B | 0.70 | 0.81 |
| Nd_2_Fe_14_B | 1.57 | 1.60 |

Supplementary Table 1. Comparison of saturation magnetization *μ*_0_*M*_s_ (T) for RE_2_Fe_14_B ferromagnets [ref 35].


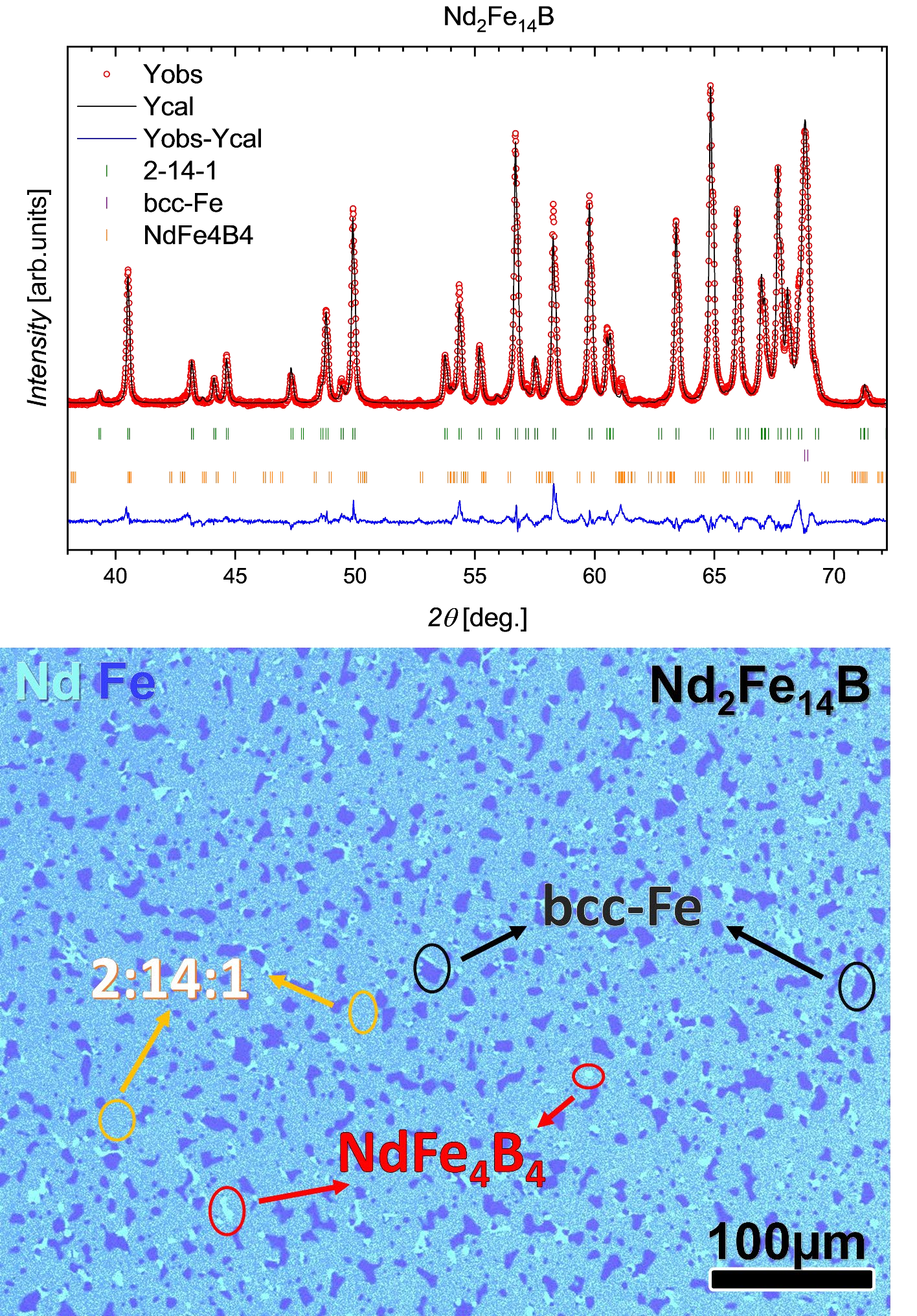
**a**

**b**

Supplementary Figure S1: (a) Rietveld analysis and (b) chemical composition analysis using SEM-EDS for the Nd_2_Fe_14_B alloys

**a**


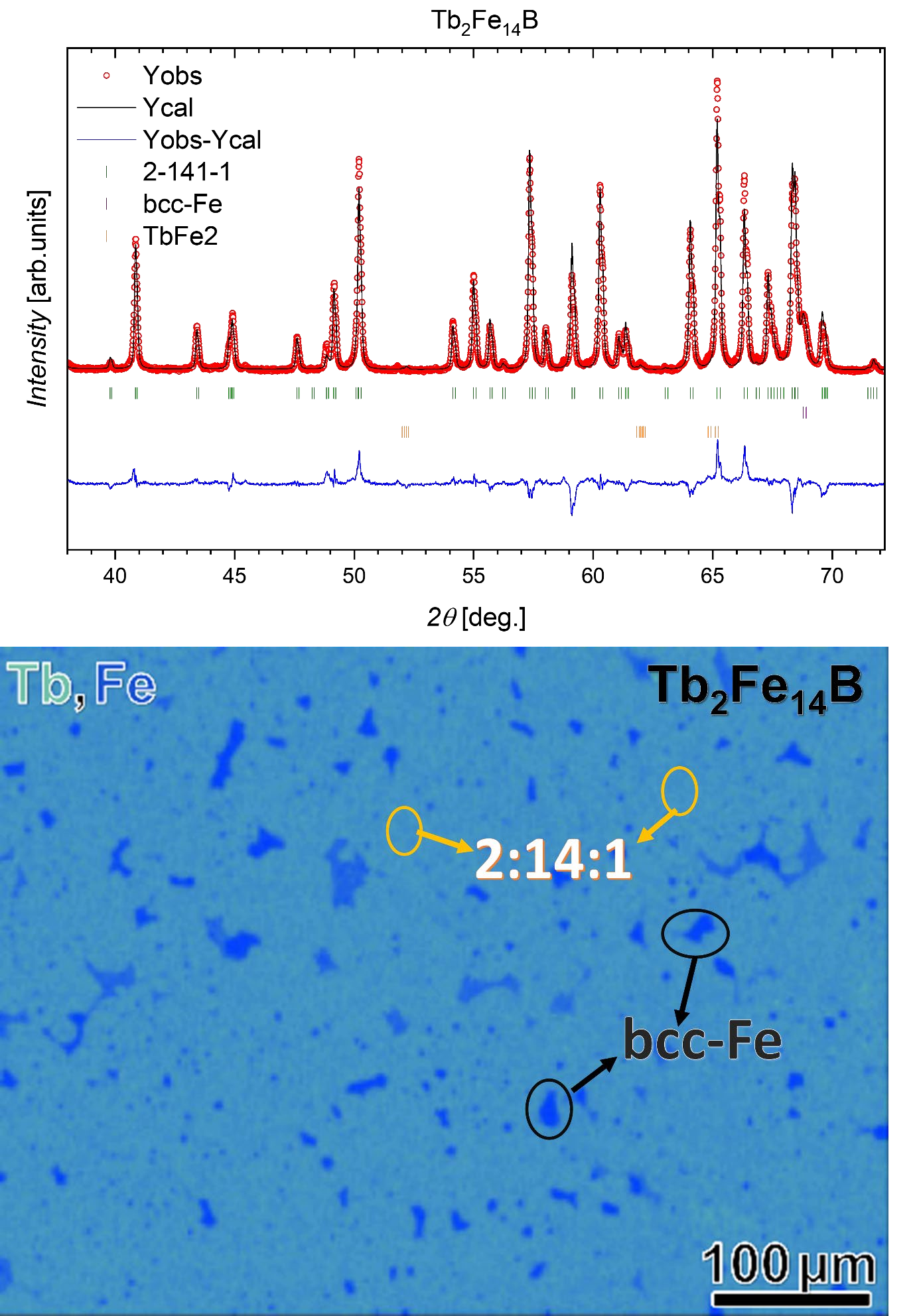
**b**

Supplementary Figure S2: (a) Rietveld analysis and (b) chemical composition analysis using SEM-EDS for the Tb_2_Fe_14_B alloys


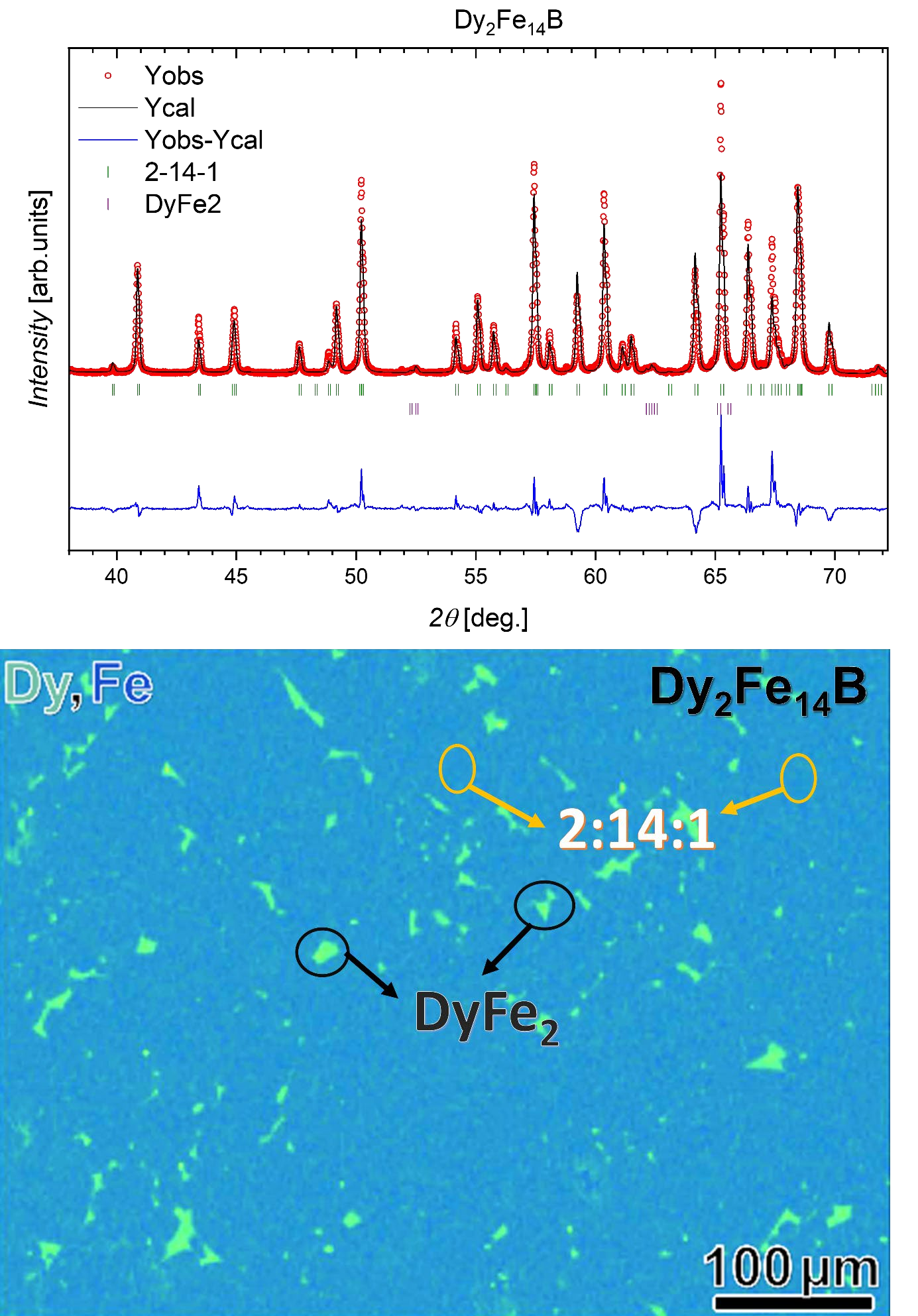
**a**

**b**

Supplementary Figure S3: (a) Rietveld analysis and (b) chemical composition analysis using SEM-EDS for the Dy_2_Fe_14_B alloys


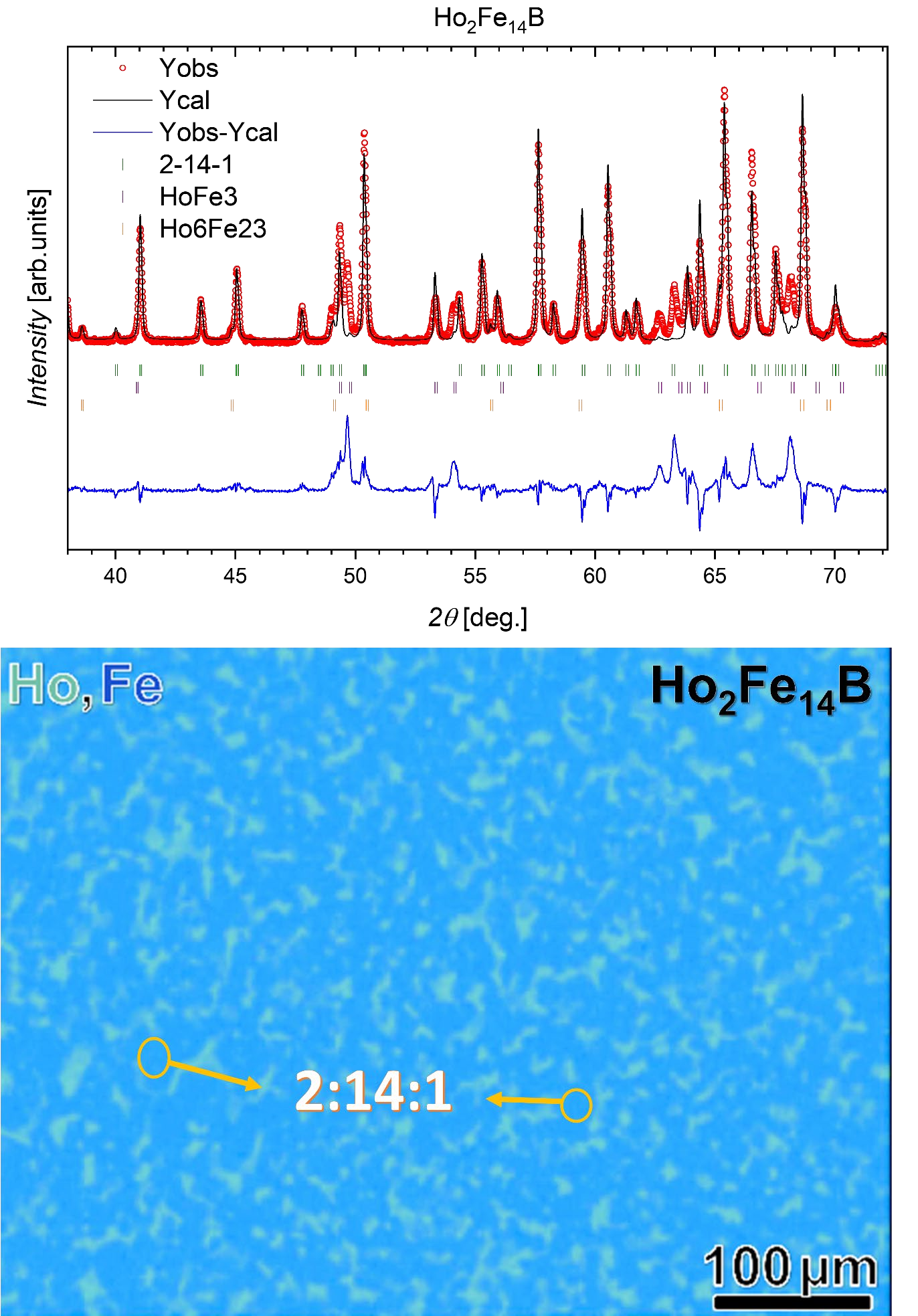
**a**

**b**

Supplementary Figure S4: (a) Rietveld analysis and (b) chemical composition analysis using SEM-EDS for the Ho_2_Fe_14_B alloys. The identification of all secondary phases in the Ho_2_Fe_14_B system remains challenging in SEM-EDS, and some of the secondary reflections found in XRD would not be identified with the existing phases.


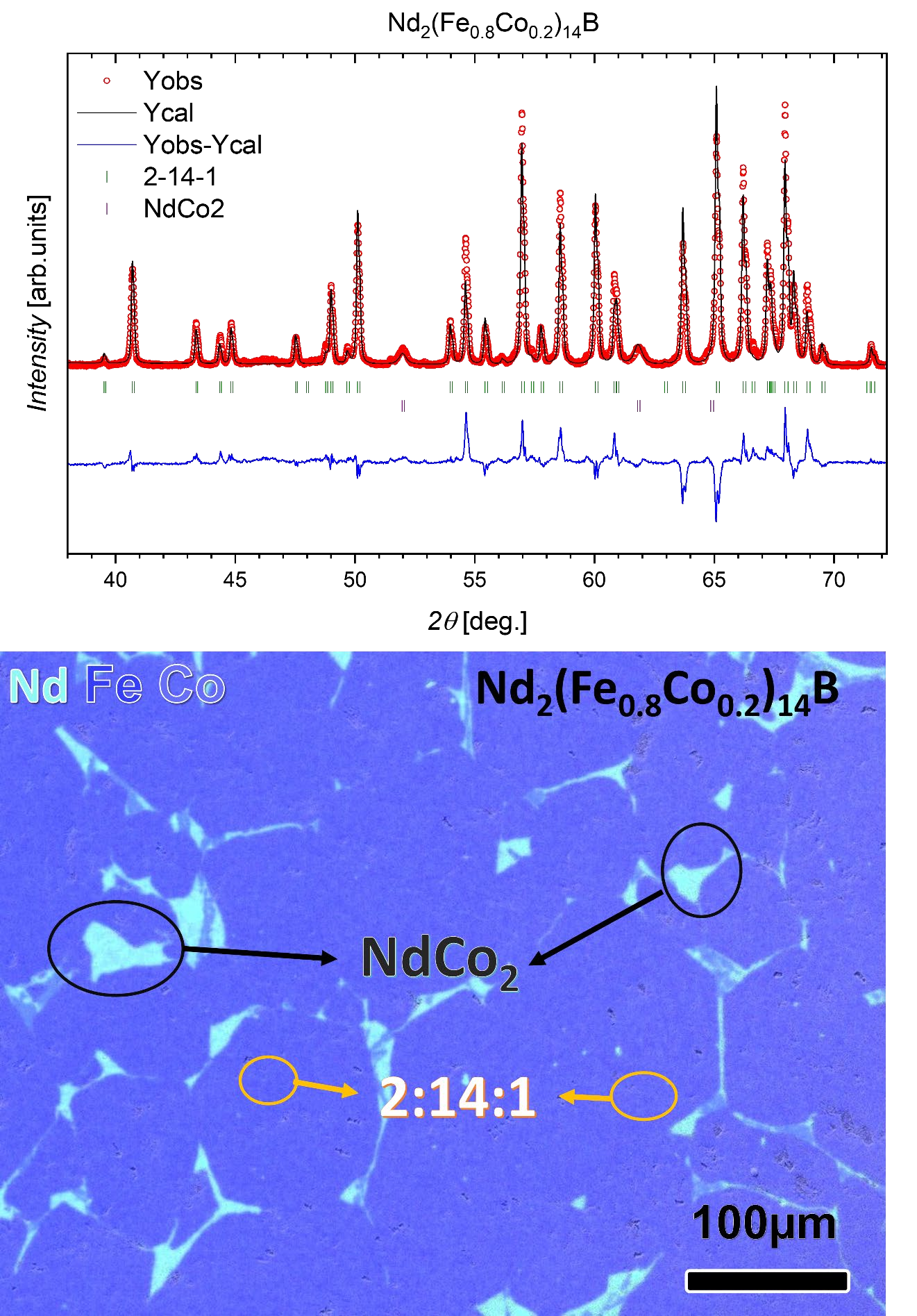
**a**

**b**

Supplementary Figure S5: (a) Rietveld analysis and (b) chemical composition analysis using SEM-EDS for the Nd_2_(Fe_0.8_Co_0.2_)_14_B alloys


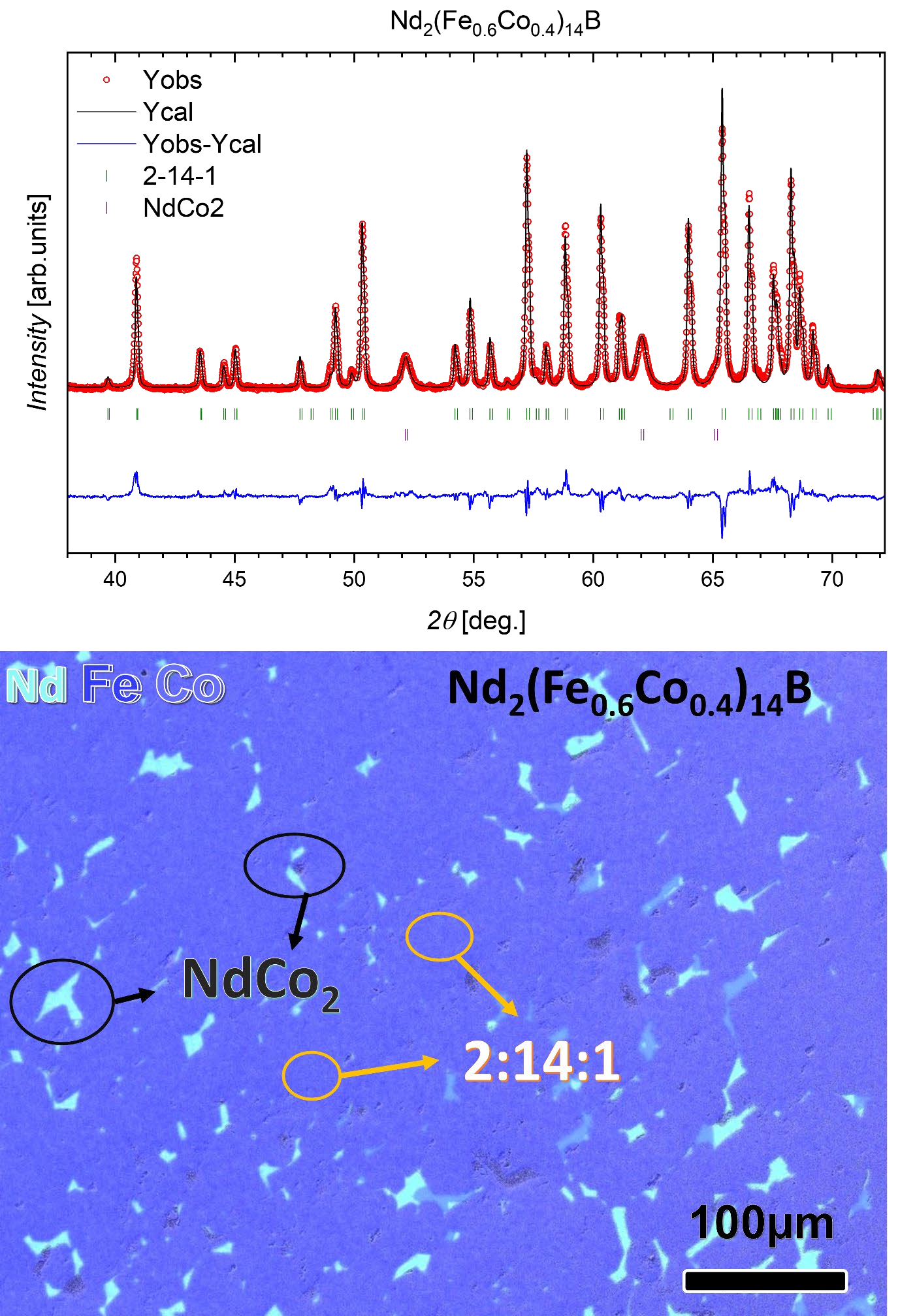
**a**

**b**

Supplementary Figure S6: (a) Rietveld analysis and (b) chemical composition analysis using SEM-EDS for the Nd_2_(Fe_0.6_Co_0.4_)_14_B alloys


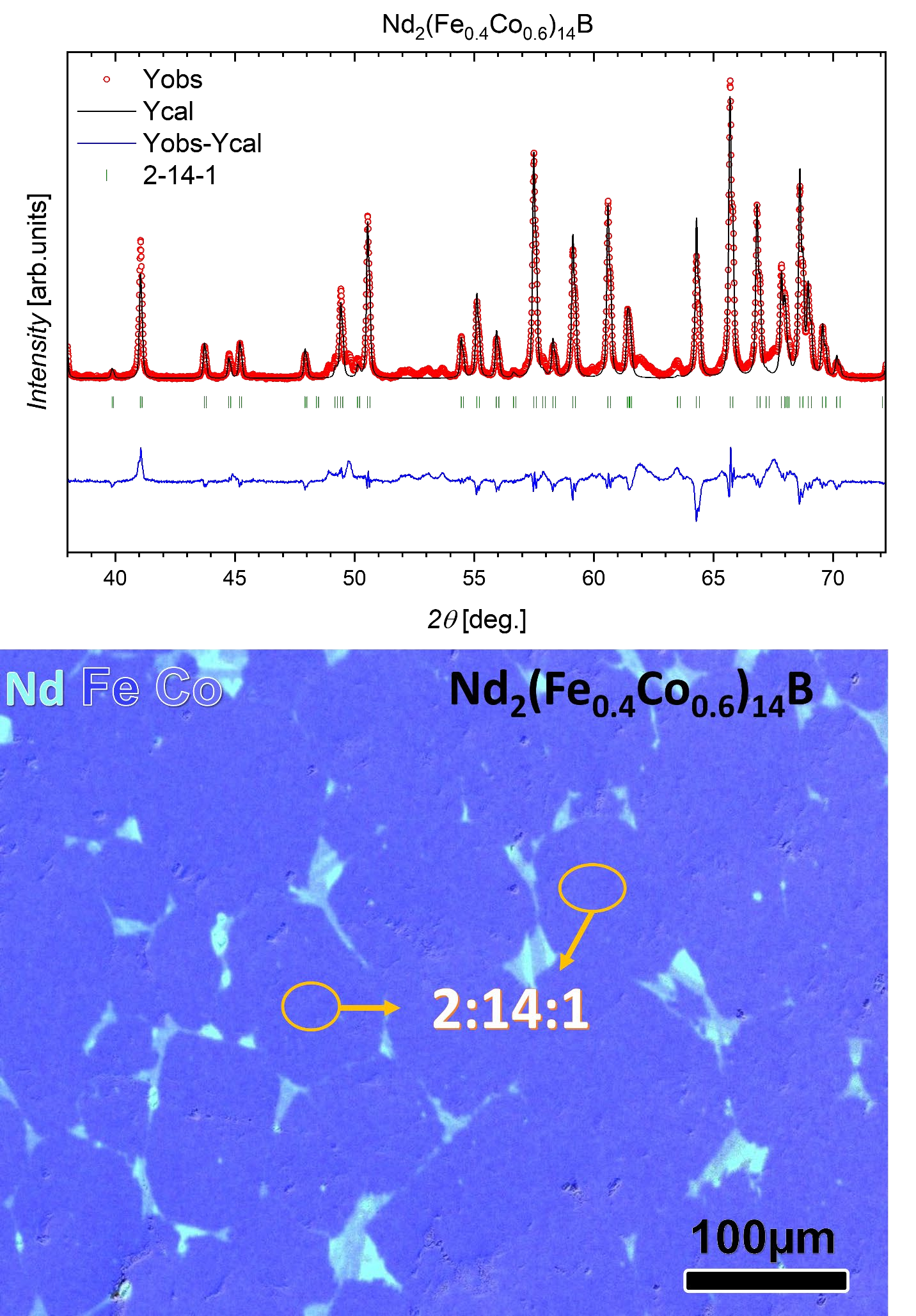
**a**

**b**

Supplementary Figure S7: (a) Rietveld analysis and (b) chemical composition analysis using SEM-EDS for the Nd_2_(Fe_0.4_Co_0.6_)_14_B alloys. The secondary phases at all triple junctions contain both RE-rich and RE-deficiency regions observed in the SEM-EDS image. Precise identification of these secondary phases remains challenging, as they are present in small volume fraction and might not be able to detectable in low-magnification SEM images, which provide more local information.


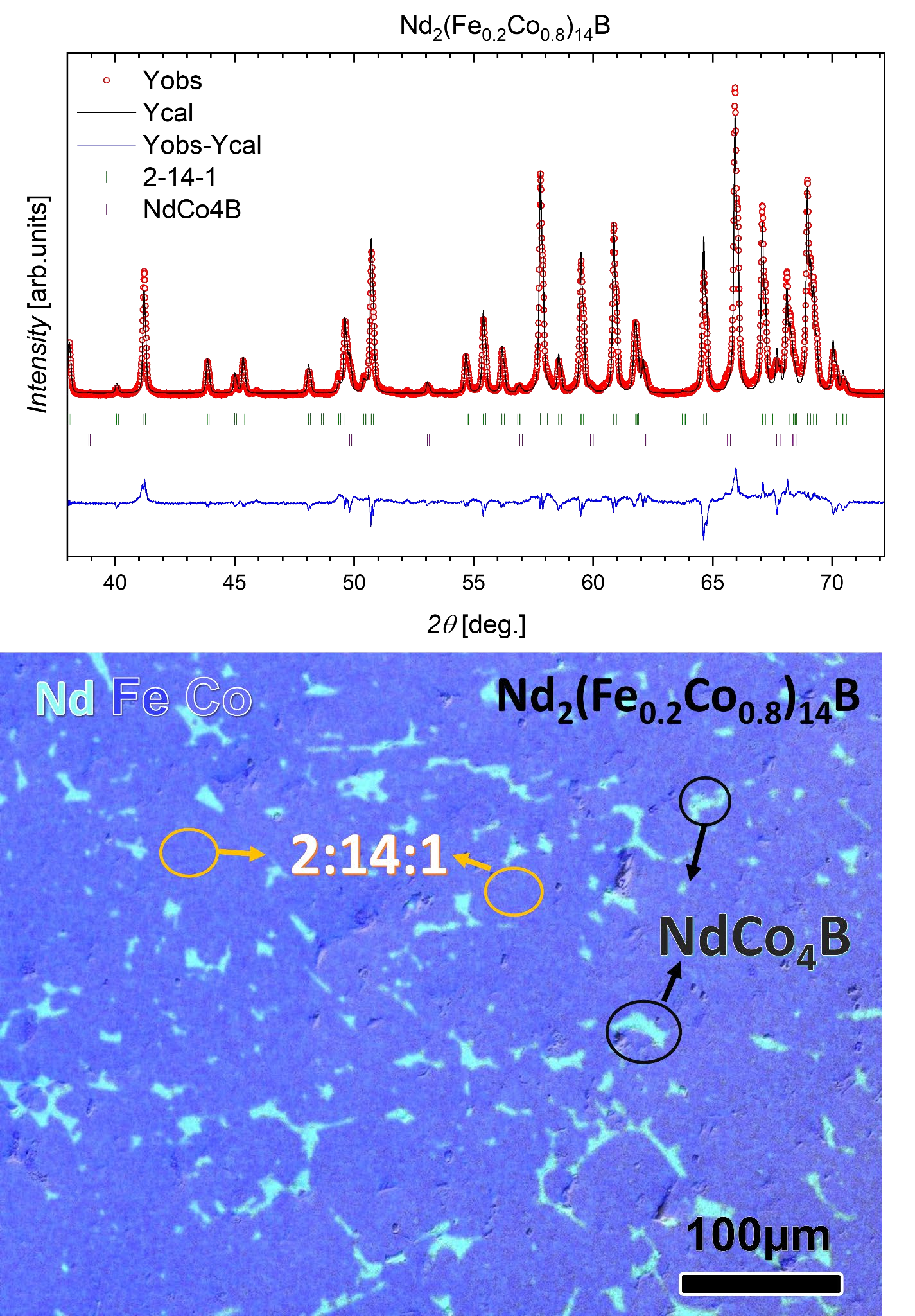
**a**

**b**

Supplementary Figure S8: (a) Rietveld analysis and (b) chemical composition analysis using SEM-EDS for the Nd_2_(Fe_0.2_Co_0.8_)_14_B alloys


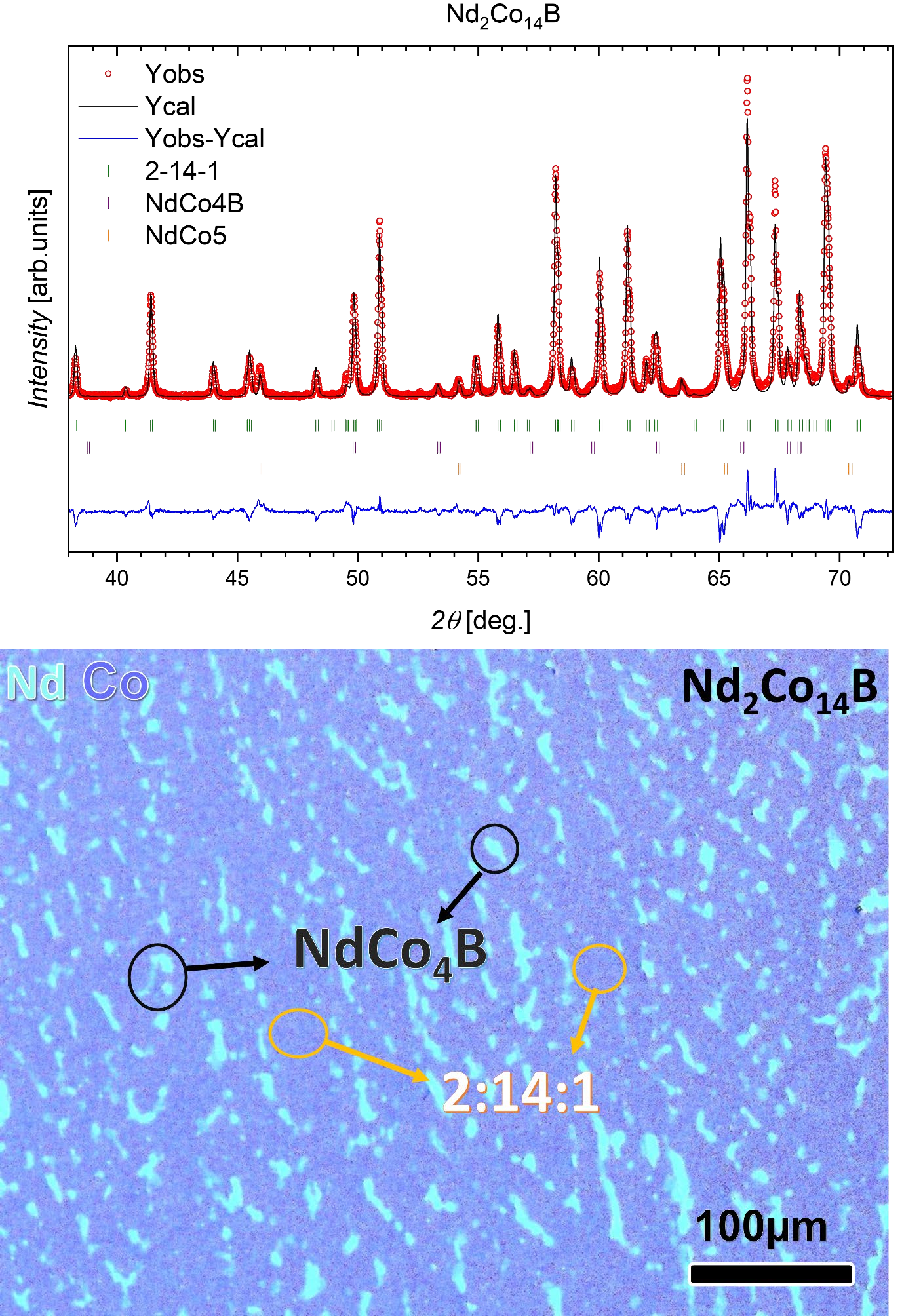
**a**

**b**

Supplementary Figure S9: (a) Rietveld analysis and (b) chemical composition analysis using SEM-EDS for the Nd_2_Co_14_B alloys


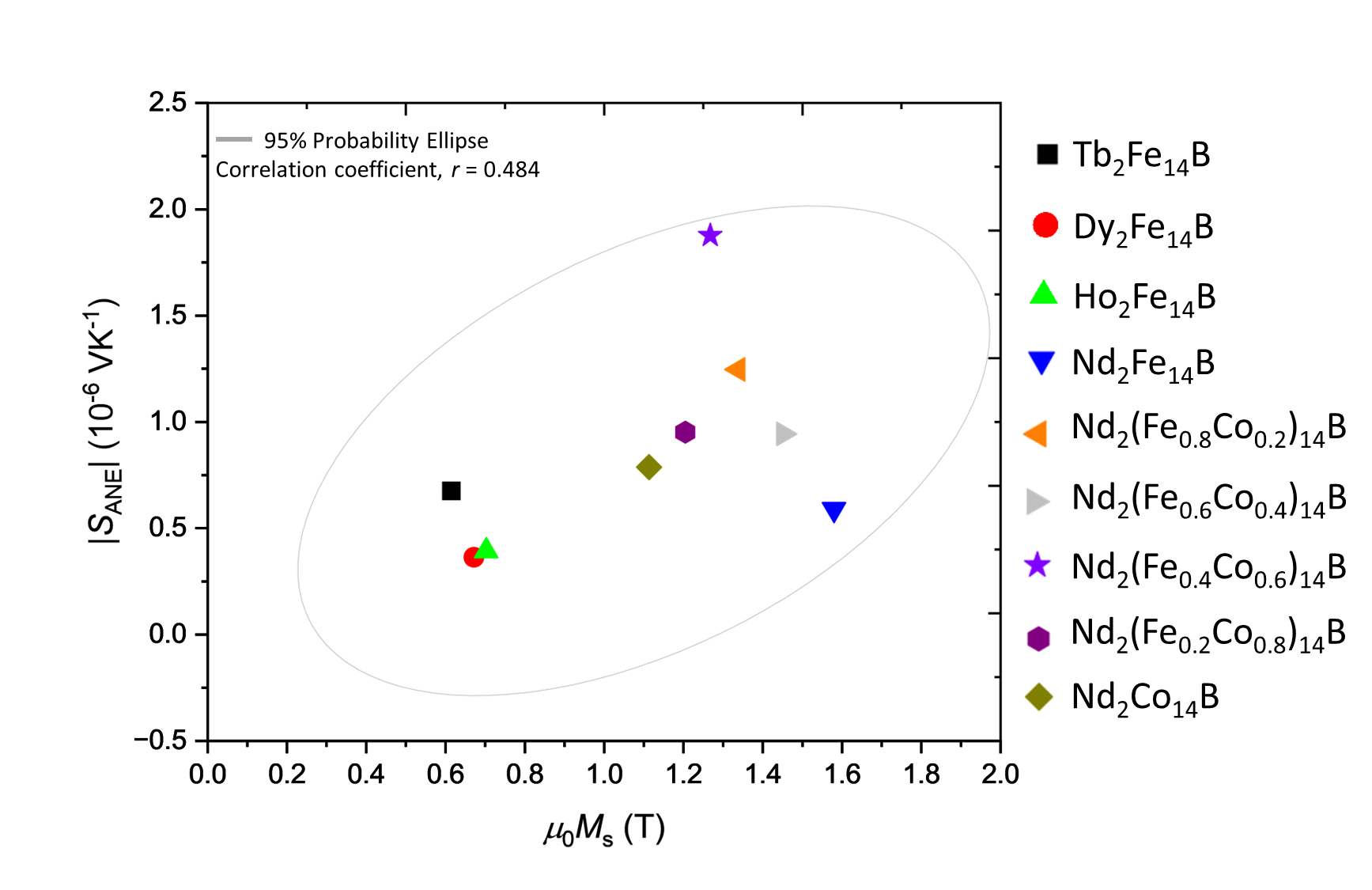


Supplementary Figure S10: Saturation magnetization *M*_s_ dependence of the absolute values of the anomalous Nernst coefficient |*S*_ANE_| for RE_2_(Fe,Co)_14_B (RE = rare-earth) alloys. The estimated correlation coefficient, *r* = 0.484 indicated the weak to moderate positive linear correlation between *M*_s_ and ANE coefficient in our work.
